# Supplementary material for: A Common Structure Underlies Low-Frequency Cortical Dynamics in Movement, Sleep, and Sedation
Source: Neuron. 2014 Sep 3;83(5):1185–99. doi: 10.1016/j.neuron.2014.07.022 (PMC4157580; doi:10.1016/j.neuron.2014.07.022)
Supplement: Document S1. Figures S1–S5 and Tables S1–S5 [file mmc1.pdf]

Neuron, Volume *83*

Supplemental Information

A Common Structure Underlies Low-Frequency Cortical Dynamics in Movement, Sleep, and Sedation

Thomas M. Hall, Felipe de Carvalho, and Andrew Jackson

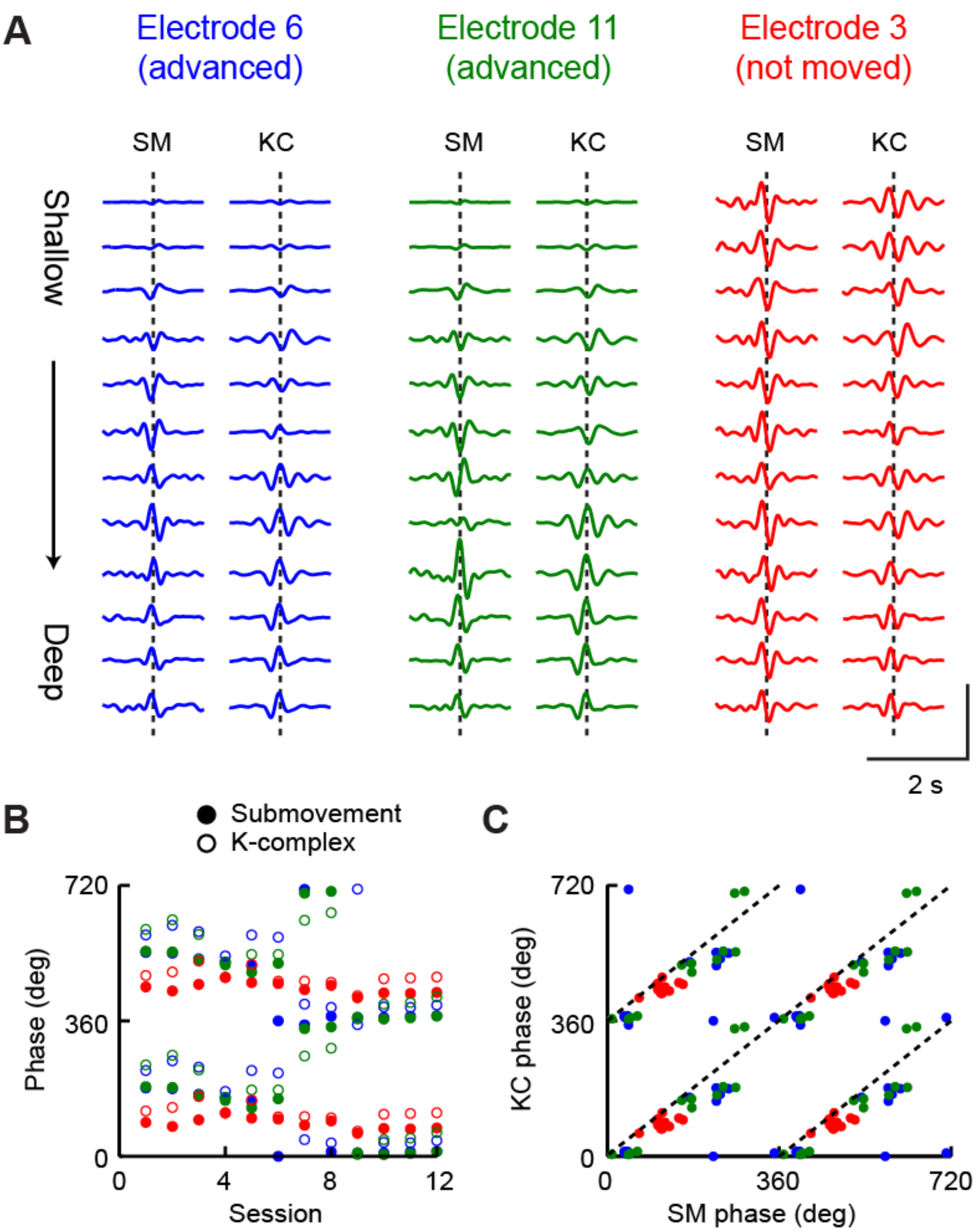

Figure S1 Related to Figure 4: Submovement- and K-complex-related LFPs share common cortical sources.

(A) Over progressive days (alternating between awake and sedated recordings), electrodes 6 and 11 were advanced from the cortical surface down through the gray matter in steps of approximately 0.5 mm. Electrode 3 was left at a fixed depth as a control. Plot shows average LFPs from these electrodes aligned to submovements (SM) and K-complexes (KC). Vertical scale: 40 µV (SM), 1000 µV (KC).

(B) Event-triggered LFP-phase for submovements (filled circles) and K-complexes (open circles) over successive sessions. Note the 180° phase shift (polarity reversal) that occurs in both submovement- and KC-triggered averages for electrode 6 (blue) and electrode 11 (green) between sessions 5-7, corresponding to a depth of approximately 3 mm. At this location we were able to record large single-units (not shown) on the same electrodes, indicating polarity reversal occurred within the grey matter. Electrode 3 (red) was not moved and no polarity inversion is seen.

(C) KC-phase plotted against SM-phase for each electrode over successive sessions. Points are color-coded according to electrode number. Across all depths the phase relative to submovements was strongly correlated with phase relative to K-complexes ($n=36$, $\rho_{CC}=0.75$, $P=8\times10^{-5}$).

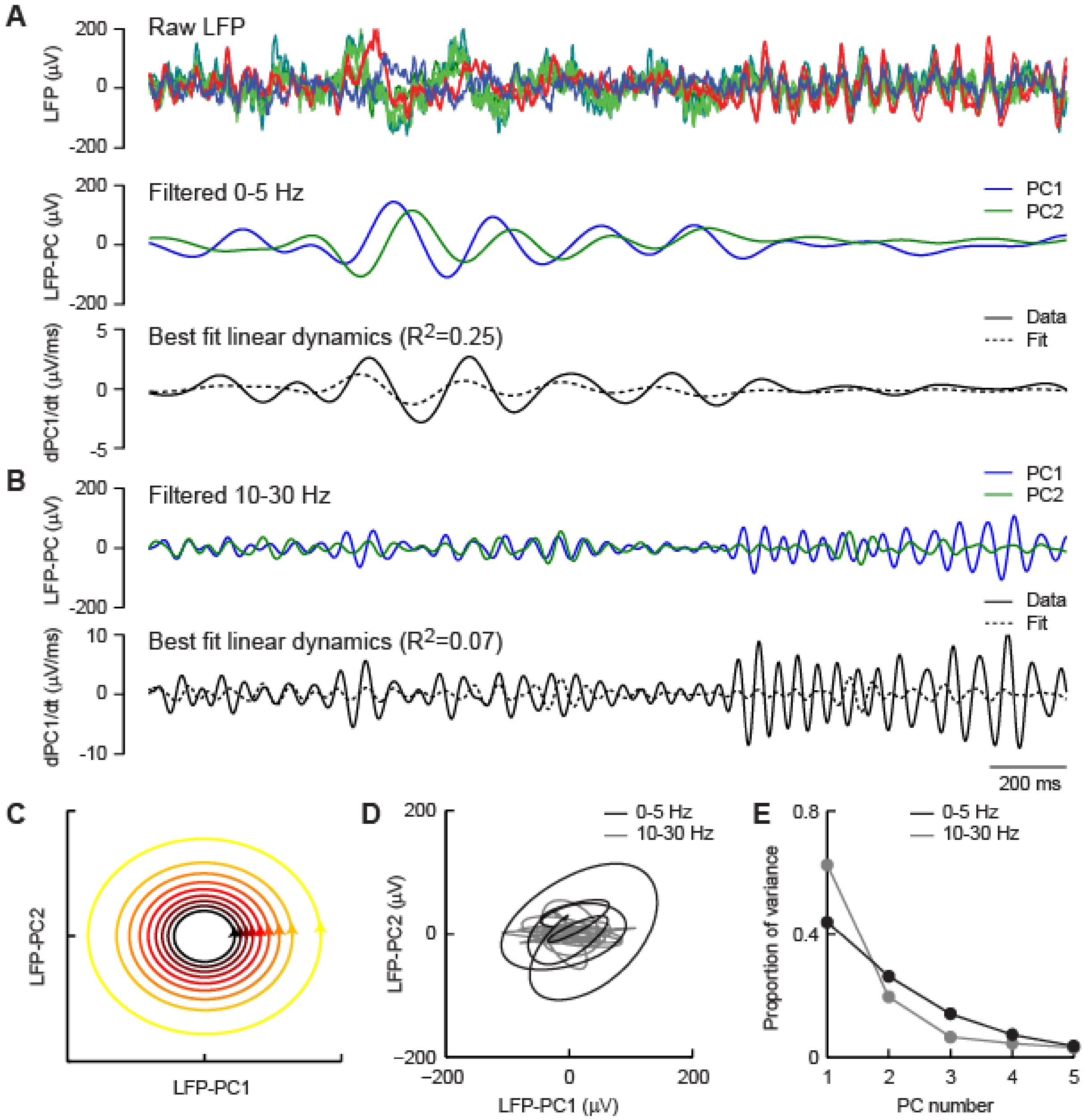

Figure S2 Related to Figure 5: A linear dynamical model fitted to LFP data in the delta and beta band.

(A) *Top:* Section of unfiltered, multichannel LFP from 9 M1 electrodes during task performance exhibiting delta and beta band oscillation. *Middle:* LFP-PCs calculated for LFP data filtered in the delta band (0-5 Hz). *Bottom:* Model fit of the time-derivative of LFP-PC1 from weighted sum of LFP-PC1 and LFP-PC2. Note that due to the consistent 90° phase-shift between PCs, the time-derivative of LFP-PC1 closely matches the negation of LFP-PC2.

(B) Equivalent analysis for same LFP data filtered in the beta band (10-30 Hz). Note that due to the lack of a consistent relationship between LFP-PCs, the time-derivative of LFP-PC1 cannot be well described by a weighted sum of LFP-PC1 and LFP-PC2.

(C) Sample closed trajectories of linear model fit to delta band LFP data.

(D) Example real LFP-PC trajectories for delta band (black) and beta band (grey).

(E) Proportion of variance captured by first five PCs in delta (black) and beta (grey) band. Note that the beta band predominantly comprises a single component (explaining ~60% of the variance), whereas the delta band signal is distributed across multiple components.

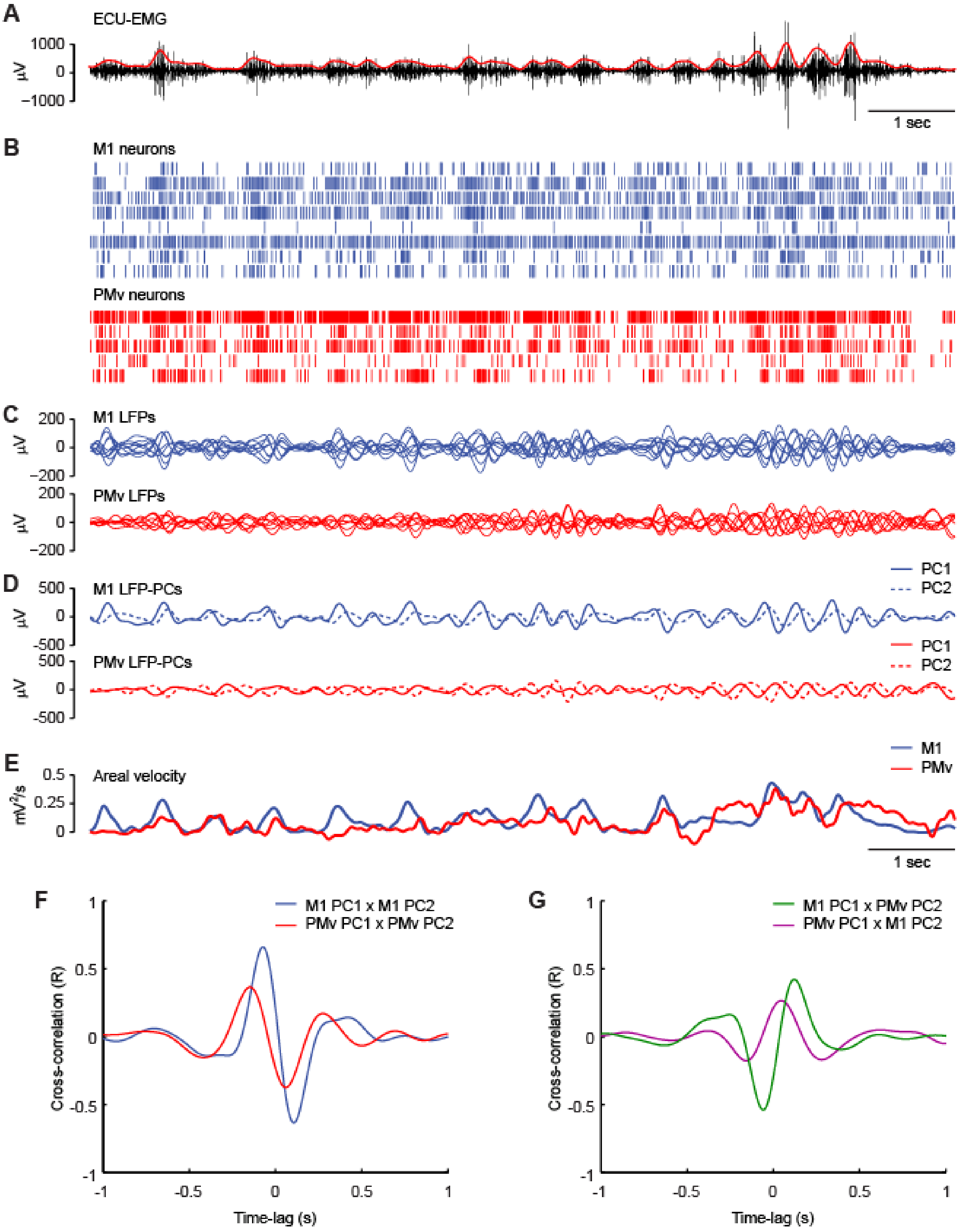

Figure S3 Related to Figure 6: M1 and PMv are co-active during free reach-to-grasp.

(A) EMG recording from *extensor carpi radialis* muscle as monkey D retrieved food rewards from small wells in a Klüver board with the arm contralateral to recording sites.

(B) Spike rasters for 8 neurons in M1 (blue) and 6 neurons in PMv (red). Note that neurons in both areas are co-active with EMG in the contralateral limb.

(C) Low-pass filtered, mean-subtracted LFPs recorded from 10 electrodes in M1 and 8 electrodes in PMv show bursts of low-frequency oscillation.

(D) LFP-PCs calculated from M1 and PMv recordings.

(E) Areal velocity in the PC plane for M1 and PMv LFPs.

(F) Cross-correlation between LFP-PCs within the same cortical area.

(G) Cross-correlation between LFP-PCs across cortical areas.

Data from monkey D, recorded at the end of the task session shown in Figure 6.

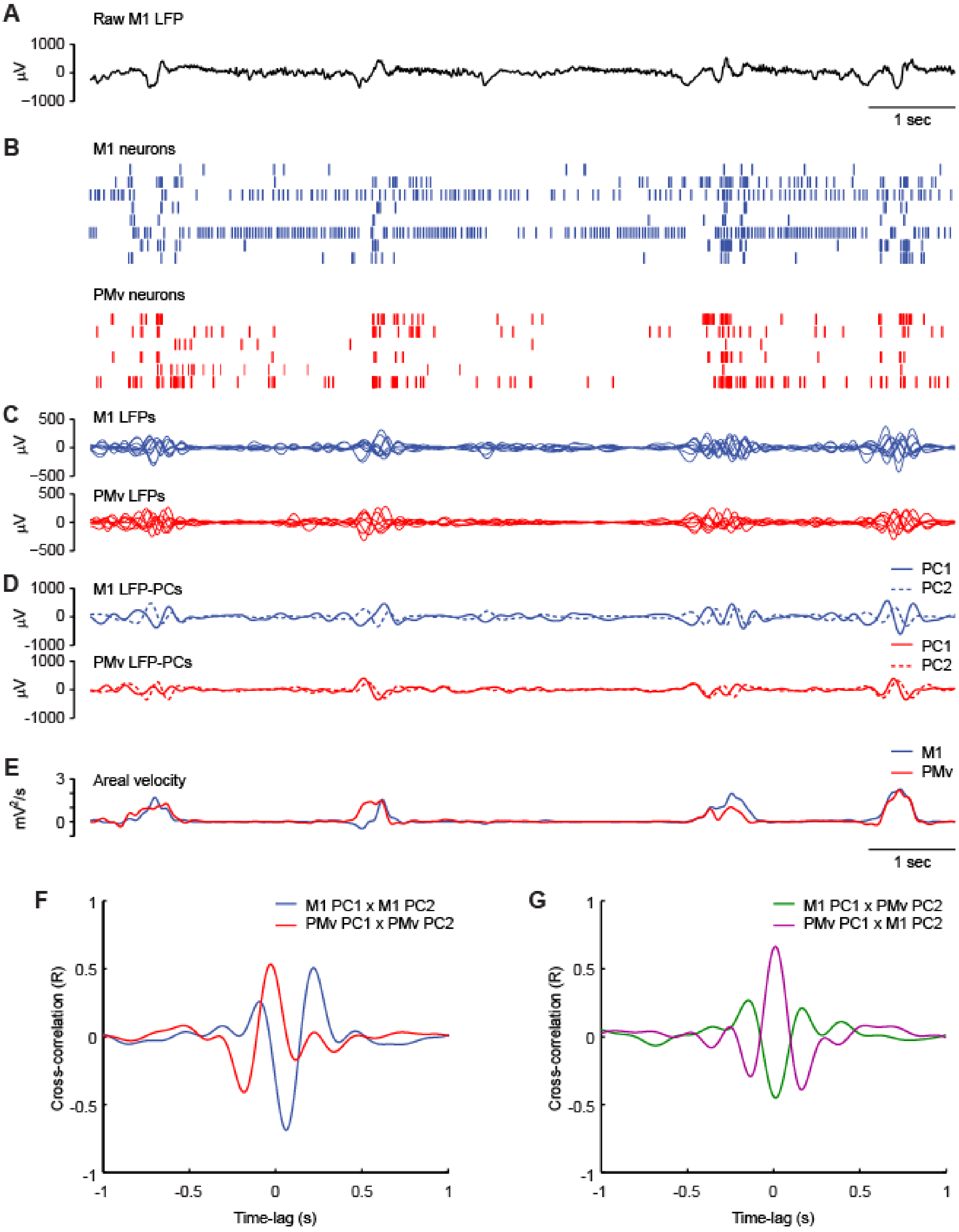

Figure S4 Related to Figure 6: M1 and PMv are co-active during K-complexes under ketamine sedation.

(A) Unfiltered LFP recording from M1 during ketamine sedation showing intermittent K-complexes.

(B) Spike rasters for 8 neurons in M1 (blue) and 6 neurons in PMv (red). Note that neurons in both areas are quiet immediately preceding the K-complex (down state) and fire maximally during its rising phase and peak (up state).

(C) Low-pass filtered, mean-subtracted LFPs recorded from 10 electrodes in M1 and 8 electrodes in PMv show bursts of low-frequency oscillation around K-complexes.

(D) LFP-PCs calculated from M1 and PMv recordings.

(E) Areal velocity in the PC plane for M1 and PMv LFPs.

(F) Cross-correlation between LFP-PCs within the same cortical area.

(G) Cross-correlation between LFP-PCs across cortical areas.

Data from monkey D, recorded following the session shown in Figure 6.

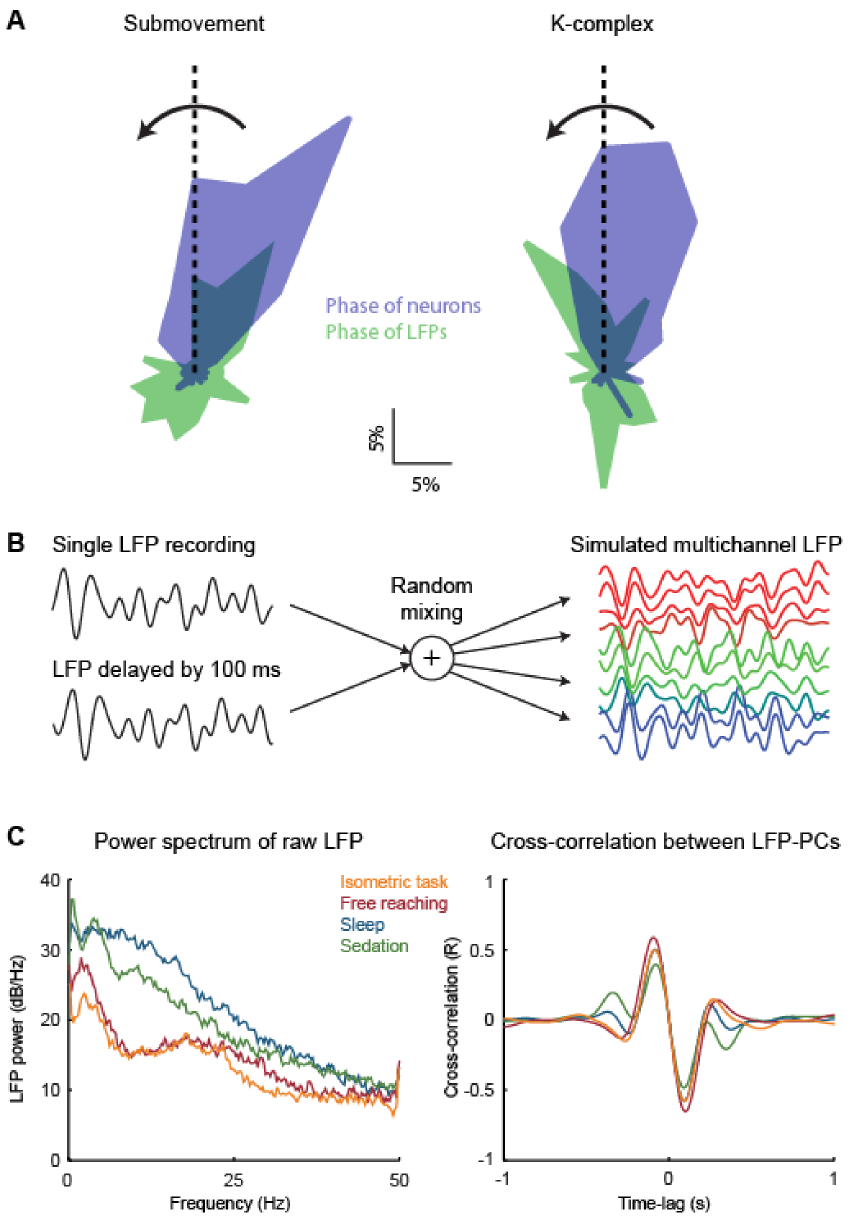

Figure S5 Related to Figure 8: Multiple phases of low-frequency oscillation are present in LFPs, while neurons fire predominantly at a single phase.

(A) Phase histogram for M1 neuronal firing (blue) and LFP (green) relative to submovements (*left*) and K-complexes (*right*). LFP data is taken from Figure 4F. Neuronal data is adapted from Figure 8. Note that the majority of M1 cells are active at a phase that slightly precedes the peak submovement speed or K-complex. By contrast, the phase of the LFP exhibits a more uniform distribution relative to each event.

(B) A simple model to explain how multiple LFP phases can arise from a mixture of underlying sources with fixed temporal delay. In this case, two LFP sources have a relative time-lag of 100 ms. Multiple LFPs are simulated by different linear mixtures of the two underlying sources.

(C) The model applied to real data. The power spectrum (*left*) is for a single channel of LFP (as in Fig. 5A). This channel was used to generate two sources, from which 10 random mixtures were drawn to simulate multichannel LFP recordings. PCA was applied to the simulated data, and the first two LFP-PCs exhibited a consistent correlation structure (*right*) that was qualitatively similar to the real data (Fig. 5B). Note that this result is relatively insensitive to the precise time delay (in this case 100 ms), since PCA finds orthogonal projections of the underlying oscillation. We speculate that the LFP captures a component of population activity that has a consistent phase-lag relative to neuronal firing. Such a component could reflect inhibitory neurons that are under-sampled in our recordings, due to their size and/or location. Alternatively, the delayed LFP component could reflect slow $GABA_B$-mediated recurrent inhibition, or synaptic currents arising from thalamo-cortical loops. Further research will be required to distinguish these possibilities.

**Monkey**	**Number of electrodes**	**Number of submovements**	**CoD** (speed)	**CoD** (direction)
D	10	1715	**0.46**	**0.07**
D	10	2043	**0.24**	0.04
D	10	1724	**0.49**	**0.17**
D	10	2049	**0.48**	**0.24**
D	10	1515	**0.45**	**0.12**
D	10	2272	**0.34**	**0.13**
R	10	914	**0.31**	**0.32**
R	10	1259	**0.04**	**0.16**
R	10	501	**0.27**	**0.23**
R	9	808	**0.22**	**0.15**
S	7	627	**0.21**	**0.12**
S	7	933	**0.17**	**0.08**
S	7	396	**0.25**	**0.12**
Average ± SD			*0.30 ± 0.13*	*0.15 ± 0.07*

Table S1 Related to Figure 2: Decoding speed and direction of individual submovements from the magnitude and direction of areal velocity vector in the 3D LFP-PC space. The average areal velocity vectors associated with all but one submovement were fitted with a parametric function of submovement speed and direction (Equ. 4,5; Experimental procedures). The speed (or direction) of the excluded submovement was then decoded from the magnitude (or orientation) of its associated areal velocity vector (Equ. 6,7). This was procedure was repeated for all submovements in the session, and the quality of the decoding was assessed using scalar (or angular) CoD (Equ. 8,9) which ranges from 0 (chance performance) to 1 (perfect decoding). The significance of individual CoD values was assessed using surrogate datasets in which the speed (or direction) was shuffled across submovements. Bold indicates P<0.05.

Monkey	Number of electrodes	ρ_{CC}
D	11	**0.63**
D	10	0.06
D	11	**0.84**
D	10	**0.85**
D	10	**0.81**
R	9	**0.90**
R	9	**0.95**
R	9	**0.86**
R	9	0.46
S	11	**0.95**
S	11	**0.93**
S	11	**0.93**
S	12	**0.56**
Average ± SD		*0.75 ± 0.25*

Table S2 Related to Figure 4. Relationship between M1 LFP phase relative to submovements (during task performance) versus phase relative to K-complexes (under sedation). Event-triggered average LFP traces were compiled for each LFP electrode separately. The LFP phase relative to each event (submovement or K-complex) was determined using a Hilbert transform. The relationship between these phases across all electrodes was quantified using the circular-circular correlation coefficient (ρ_{CC}). Bold indicates P<0.05.

Monkey	Delta band (0-5 Hz)			Beta band (10-30 Hz)		
	Task fit	Task-sedation generalization	Sedation fit	Task fit	Task-sedation generalization	Sedation fit
D	0.31	0.41	0.50	0.14	-0.08	0.02
D	0.33	0.28	0.44	0.02	0.04	0.07
D	0.22	0.26	0.43	0.03	-0.05	0.002
D	0.31	0.14	0.32	0.13	-0.08	0.08
D	0.22	0.07	0.15	0.13	-0.09	0.10
R	0.14	0.03	0.04	0.01	0.01	0.007
R	0.27	0.27	0.27	0.01	0.01	0.008
R	0.09	0.12	0.16	0.06	-0.11	0.0009
R	0.15	0.12	0.14	0.08	-0.11	0.003
S	0.13	0.16	0.18	0.01	-0.02	0.001
S	0.17	0.24	0.25	0.06	-0.11	0.001
S	0.16	0.23	0.26	0.04	-0.05	0.002
Average (SD)	*0.20 (0.08)*	*0.20 (0.10)*	*0.26 (0.13)*	*0.06 (0.05)*	*-0.05 (0.05)*	*0.02 (0.03)*

Table S3 Related to Figure 5: Linear dynamical model fit to 2D LFP-PC data in delta and beta bands for task performance and ketamine sedation. For each frequency band, the first column shows quality of fit (measured by CoD; Equ. 10) when model parameters were fitted to and tested on the same data, recorded during performance of the isometric task (Task fit). The second column shows how well the same model parameters that best-fit the task data were able to fit the data recorded on a different day during ketamine sedation (Task-sedation generalization). The third column shows the performance of the model using parameters that best-fit the sedation data when tested on the same data (Sedation fit). The data are plotted in Figure 5D.

Monkey	Delta band (0-5 Hz)			Beta band (10-30 Hz)		
	Task fit	Task-sleep generalization	Sleep fit	Task fit	Task-sleep generalization	Sleep fit
D	0.26	0.11	0.14	0.14	-0.11	0.03
D	0.24	0.24	0.26	0.14	-0.12	0.04
D	0.28	0.15	0.21	0.13	-0.13	0.02
D	0.22	0.17	0.18	0.13	0.01	0.09
S	0.18	0.15	0.18	0.02	-0.01	0.006
S	0.13	0.20	0.22	0.01	0.05	0.09
S	0.17	0.12	0.14	0.06	0.11	0.13
S	0.16	0.13	0.14	0.04	0.09	0.11
S	0.15	0.11	0.12	0.03	0.07	0.09
Average (SD)	*0.20 (0.05)*	*0.15 (0.04)*	*0.18 (0.04)*	*0.08 (0.05)*	*-0.004 (0.09)*	*0.07 (0.04)*

Table S4 Related to Figure 5: Linear dynamical model fit to 2D LFP-PC data in delta and beta bands for task performance and natural sleep. For each frequency band, the first column shows quality of fit (measured by CoD; Equ. 10) when model parameters were fitted to and tested on the same data, recorded during performance of the isometric task (Task fit). The second column shows how well the same model parameters that best-fit the task data were able to fit the data recorded during natural sleep at the end of the session (Task-sleep generalization). The third column shows the performance of the model using parameters that best-fit the sleep data when tested on the same data (Sleep fit). The data are plotted in Figure 5E.

Monkey	Delta band (0-5 Hz)			Beta band (10-30 Hz)		
	Task fit	Task-free generalization	Free fit	Task fit	Task-free generalization	Free fit
D	0.33	0.17	0.23	0.11	0.09	0.10
D	0.26	0.25	0.30	0.16	0.14	0.14
D	0.31	0.16	0.25	0.15	0.11	0.11
D	0.28	0.17	0.26	0.13	0.12	0.12
D	0.22	0.20	0.25	0.13	0.11	0.12
D	0.23	0.30	0.39	0.14	0.12	0.13
S	0.13	0.14	0.14	0.03	0.01	0.01
S	0.12	0.13	0.14	0.04	0.01	0.02
S	0.11	0.15	0.16	0.02	0.02	0.02
S	0.11	0.15	0.16	0.02	0.01	0.01
Average (SD)	*0.21 (0.08)*	*0.18 (0.05)*	*0.23 (0.08)*	*0.09 (0.05)*	*0.05 (0.05)*	*0.08 (0.05)*

Table S5 Related to Figure 5: Linear dynamical model fit to 2D LFP-PC data in delta and beta bands for task performance and free reaching for food in a Klüver board. For each frequency band, the first column shows quality of fit (measured by CoD; Equ. 10) when model parameters were fitted to and tested on the same data, recorded during performance of the isometric task (Task fit). The second column shows how well the same model parameters that best-fit the task data were able to fit the data recorded during free reaching at the end of the session (Task-free generalization). The third column shows the performance of the model using parameters that best-fit the free reaching data when tested on the same data (Free fit). The data are plotted in Figure 5F.

Supplementary Movies

Movie S1 Related to Figure 1: Sample M1 LFP recordings and M1 LFP-PC trajectories during torque tracking in real-time. *Top left*: task display with cursor (yellow) and target (red). *Bottom*: low-pass filtered LFP data. *Top right*: Instantaneous LFP-PCs projected onto the 2D plane.

Movie S2 Related to Figure 2: Animation of submovement-triggered average 2D LFP-PC trajectories divided according to peak cursor speed for monkey D and monkey R. Trajectories are shown from 200ms before to 200ms after the midpoint of each submovement (indicated by filled circles). Trials are sorted and color-coded according to cursor speed, as in Fig. 2F,I. Animation runs three times.

Movie S3 Related to Figure 2: Animation of submovement-triggered average 3D LFP-PC trajectories divided according to submovement direction. Trajectories are shown from 200ms before to 200ms after the midpoint of each submovement (indicated by filled circles). Trials are sorted and color-coded according to direction of movement, as in Fig. 2L,M. Thick lines indicate orientation of areal velocity vectors.

Movie S4 Related to Figure 5: Motor cortical LFP dynamics under different wake/sleep/sedation states in real-time. *Right*: Low-pass filtered LFP data in real-time during isometric movement, natural sleep and ketamine sedation. *Left*: Instantaneous projection onto PC plane (determined from data recorded during task performance). Note that the axes for movement data have been expanded x4 to compensate for the smaller amplitude of movement-related LFP relative to sleep and sedation.

Movie S5 Related to Figure 8: Animation of spike-triggered average of LFP-PC trajectories. Trajectories are shown from 200ms before to 200ms after the time of spikes from M1 (blue) and PMv (red), as in Fig. 8. Top row shows M1 LFPs and bottom row shows PMv LFPs. Different columns correspond to different behavioural states (isometric task, free reaching, natural sleep and ketamine sedation). In all cases the data are projected onto the PC plane determined from recordings during task performance. Animation runs three times.
