# Supplementary material for: A Common Structure Underlies Low-Frequency Cortical Dynamics in Movement, Sleep, and Sedation
Source: Neuron. 2014 Sep 3;83(5):1185–99. doi: 10.1016/j.neuron.2014.07.022 (PMC4157580; doi:10.1016/j.neuron.2014.07.022)
Supplement: Document S2. Article plus Supplemental Information [file mmc7.pdf]

A Common Structure Underlies Low-Frequency Cortical Dynamics in Movement, Sleep, and Sedation

Thomas M. Hall,[1] Felipe de Carvalho,[1] and Andrew Jackson[1,*]
[1]Institute of Neuroscience, Newcastle University, Framlington Place, Newcastle NE2 4HH, UK
*Correspondence: andrew.jackson@ncl.ac.uk
http://dx.doi.org/10.1016/j.neuron.2014.07.022
This is an open access article under the CC BY license (http://creativecommons.org/licenses/by/3.0/).

SUMMARY

Upper-limb movements are often composed of regular submovements, and neural correlates of submovement frequencies between 1 and 4 Hz have been found in the motor cortex. The temporal profile of movements is usually assumed to be determined by extrinsic factors such as limb biomechanics and feedback delays, but another possibility is that an intrinsic rhythmicity contributes to low frequencies in behavior. We used multielectrode recordings in monkeys performing an isometric movement task to reveal cyclic activity in primary motor cortex locked to submovements, and a distinct oscillation in premotor cortex. During ketamine sedation and natural sleep, cortical activity traversed similar cycles and became synchronized across areas. Because the same cortical dynamics are coupled to submovements and also observed in the absence of behavior, we conclude that the motor networks controlling the upper limb exhibit an intrinsic periodicity at submovement frequencies that is reflected in the speed profile of movements.

INTRODUCTION

Movement requires coordinating dynamic patterns of activity across multiple muscles. Many simple rhythmic behaviors are controlled by specialized central pattern generator (CPG) networks in the spinal cord or brainstem with intrinsic oscillatory characteristics (Grillner, 2006; Kiehn, 2006). However, goal-directed upper-limb movements under cortical control can also exhibit rhythmicity. When tracking a moving target, trajectories are composed of multiple submovements (Craik, 1947) at a frequency (usually one to four per second) that is largely independent of movement speed (Miall et al., 1986; Roitman et al., 2004; Pasalar et al., 2005; Selen et al., 2006). An oscillation at around 3 Hz has also been reported in the kinematics of finger-tracking movements (McAuley et al., 1999). The motor cortical electroencephalogram (EEG) is phase locked to submovements (Dipietro et al., 2011), and coherence spectra between the magnetoencephalogram (MEG) and movement speed show peaks around 3 Hz during visuomotor tracking (Jerbi et al., 2007). Brain-machine interface (BMI) studies have found low-frequency bands to be particularly informative for decoding direction from local field potentials (LFPs; Rickert et al., 2005; Bansal et al., 2011), MEGs (Waldert et al., 2008), and EEGs (Waldert et al., 2008; Bradberry et al., 2010; but see Antelis et al., 2013).

It has been argued that submovements reflect intermittent corrections driven by visual feedback of errors (Craik, 1947; Miall et al., 1986, 1993) and that their frequency should therefore be determined by extrinsic factors such as feedback loop delays. In support of this "extrinsic hypothesis," submovements are locked to eye movements (McAuley et al., 1999) and often disappear in the absence of vision (Miall et al., 1993; McAuley et al., 1999; but see Doeringer and Hogan, 1998), whereas the introduction of artificial feedback delays alters their frequency (Miall et al., 1986; Miall and Jackson, 2006). Nevertheless, submovements are not restricted to tracking tasks, and a natural rhythmicity is observed across diverse upper-limb behaviors (Kunesch et al., 1989) including self-paced isometric drawing (Massey et al., 1992) and finger tapping (Schöner and Kelso, 1988). Moreover, low-frequency cortical oscillations have long been associated with slow-wave sleep, when large K complex potentials signifying transitions from down to up states of the cortex (Colrain, 2005; Cash et al., 2009) are accompanied by bursts of activity in the delta (1–4 Hz)-frequency range (Amzica and Steriade, 1997). At least two mechanisms contribute to these delta oscillations: intrinsic currents that cause bursting patterns in thalamic relay cells (Amzica et al., 1992; Destexhe and Sejnowski, 2003) and a second, purely cortical circuit (Amzica and Steriade, 1998; Carracedo et al., 2013).

Therefore, it remains possible that oscillatory properties of cortical (and perhaps thalamic) circuits contribute to low-frequency rhythms in movement, functioning much like a CPG. Recently, this "intrinsic hypothesis" has been proposed to explain the complex, multiphasic profiles of motor cortical firing rates observed during reaching (Churchland et al., 2012; Shenoy et al., 2013). The high-dimensional neural state was projected onto a plane revealing low-frequency cycles, even though movements in this case were not overtly rhythmic. It was proposed that this dynamical structure reflects "an engine of movement" (Churchland et al., 2012), and could be reproduced by a recurrent neural network model trained to generate muscle patterns given static initial inputs and no sensory feedback (Shenoy et al., 2013).

The intrinsic hypothesis suggests that low-frequency cortical dynamics may be preserved across different movements and resemble spontaneous delta oscillations during sleep.

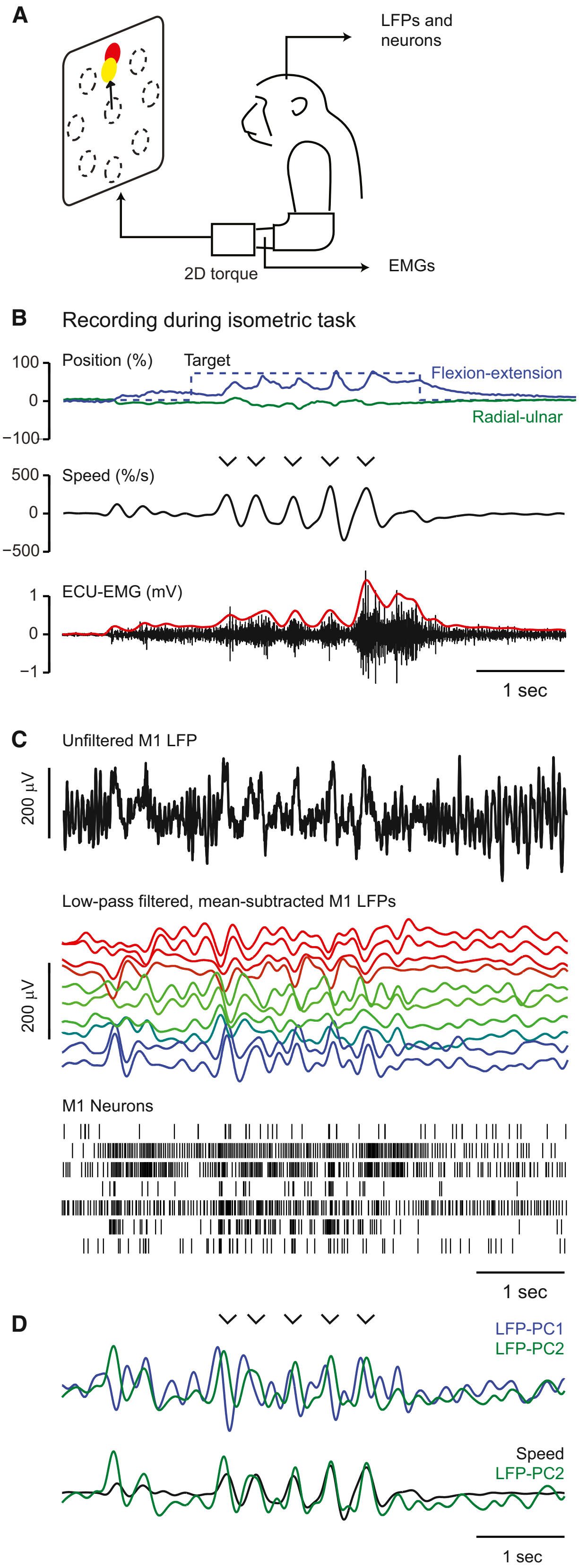

Figure 1. Low-Frequency Cortical Dynamics during Isometric Task Performance

(A) Schematic of isometric wrist-torque task.

(B) Top: position of cursor (solid lines) and target (dashed lines) during a typical trial. Middle: cursor speed (time derivative of radial position from the origin). Bottom: raw (black) and rectified, smoothed (red; not to scale) EMG from a wrist extensor muscle, extensor carpi ulnaris (ECU). Five submovements occurred during this trial, indicated by arrowheads.

(C) Top: unfiltered, surface-referenced LFP from a representative electrode in M1 during task performance. Middle: low-pass-filtered, mean-subtracted LFP from all electrodes in the M1 array, ordered and color coded according to phase relative to submovements. The bottom trace (blue) corresponds to the unfiltered signal shown at the top (black). Bottom: spike rasters for seven M1 neurons.

(D) Top: first two principal components (LFP-PCs) calculated from low-pass-filtered, mean-subtracted M1 LFP. Bottom: second LFP-PC and speed profile overlaid.

Data are from monkey D. See also Movie S1.

Therefore, we compared motor cortex activity in monkeys during an isometric movement task, while retrieving food from a Klüver board, during natural sleep, and under ketamine sedation. We found clear evidence for a common LFP correlation structure that could be explained by a single model of 3 Hz oscillatory dynamics underlying all behavioral states. Our results thereby unify two previously unrelated phenomena: low-frequency structure in movement kinematics and low-frequency oscillations during slow-wave sleep, providing a new insight into how the dynamics of cortical networks influence complex upper-limb behaviors.

RESULTS

Isometric Center-Out Wrist Movements Are Composed of Rhythmic Submovements

Three monkeys controlled the 2D position of a cursor with isometric wrist torque to acquire targets in a center-out fashion (Figure 1A). Similar to isometric trajectories in humans (Massey et al., 1992), the movements made by the monkeys were often composed of multiple, regular submovements. The representative single trial shown in Figure 1B shows five submovements between the go cue and successful acquisition of the target, appearing as peaks in the radial speed of the cursor. The distribution of intersubmovement intervals (Figure 2A), as well as their autocorrelation structure (Figure 2B), revealed a tendency for submovements to occur rhythmically at a frequency of around 3 Hz in all three animals. Movement intermittency was also evident in the electromyogram (EMG) from wrist muscles involved in the task, with a peak in coherence between radial position and rectified EMG at 3 Hz (Figure 2C).

Low-Frequency LFP Oscillations Are Phase Locked to Submovements

LFPs from multiple electrodes in primary motor cortex (M1) were low-pass filtered and mean referenced, revealing slow oscillations with a phase that varied across electrodes (Figure 1C). Principal component analysis (PCA) reduced the LFP to two principal components (LFP-PCs; Figure 1D) capturing orthogonal projections of the dominant oscillatory mode. There was a striking correlation between LFP-PCs and submovements, evident even in

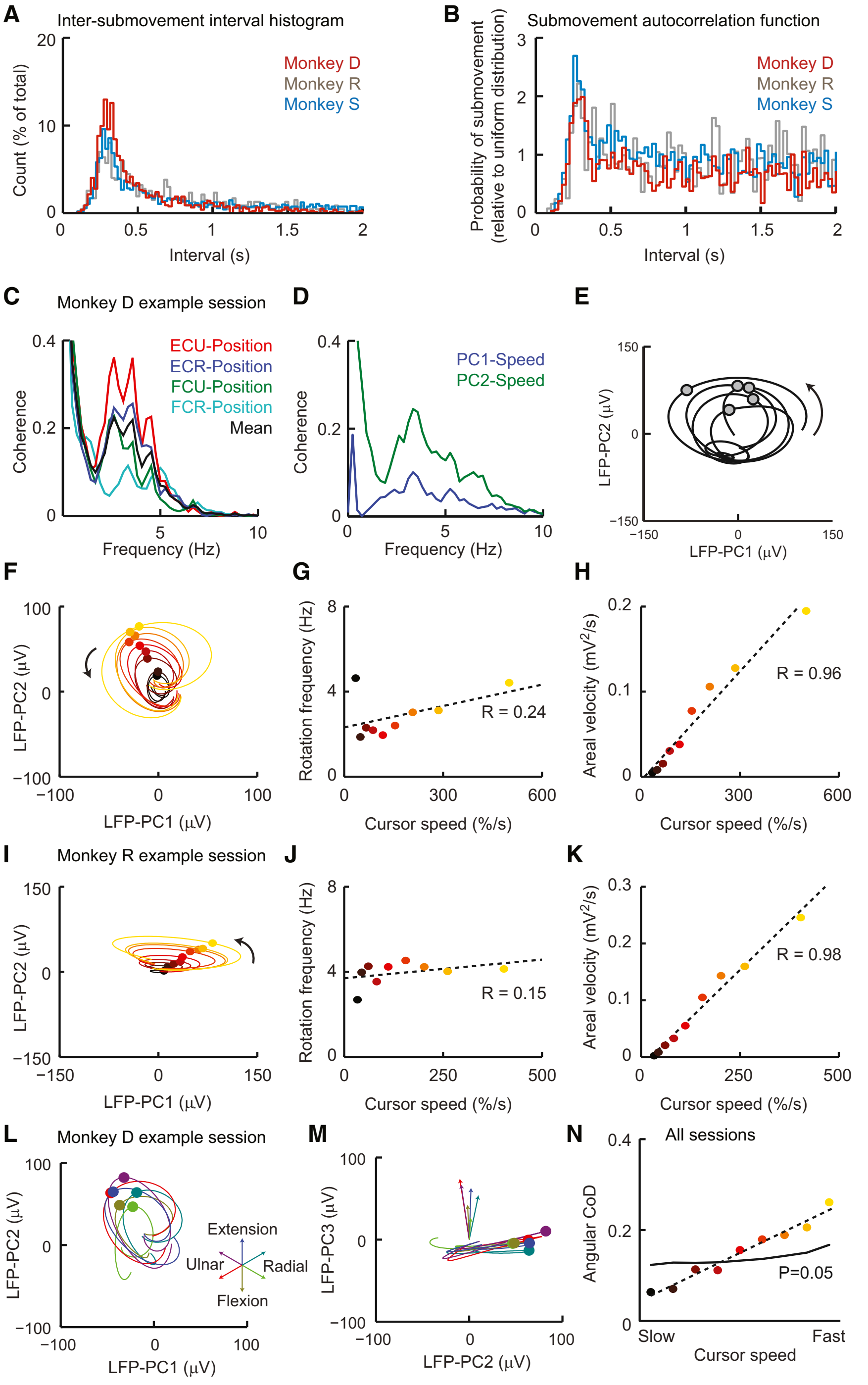

Figure 2. Relationship between LFP-PCs and Movement Kinematics

(A) Intersubmovement interval histograms for representative sessions in all three animals.

(B) Autocorrelation histogram of intervals between all pairs of submovements in the same trial (between go cue and end of successful hold). Histograms are normalized by the interval distribution expected for a uniform (Poisson) distribution with the same rate. The peak at around 300 ms reveals the underlying rhythmicity of submovements.

(C) Coherence spectra between radial cursor position and rectified EMG from four wrist muscles: extensor carpi ulnaris (ECU), extensor carpi radialis (ECR), flexor carpi ulnaris (FCU), and flexor carpi radialis (FCR). Data are from monkey D comprising 320 trials with 2,063 submovements.

(D) Coherence spectra between LFP-PCs and radial cursor speed across the same session.

(E) LFP-PC trajectory for 2 s of the representative trial shown in Figure 1. Circles indicate times of peak cursor speed.

(F) LFP-PC trajectories aligned to peak speed of submovements and averaged across nine equal-sized groups sorted by peak cursor speed. Trajectories are plotted for 200 ms on either side of time of peak speed (indicated by circles) and color coded from black to red to yellow according to cursor speed.

(G) Rotational frequency of average trajectories for different submovement speeds, calculated at the time of peak cursor speed.

(H) Areal velocity (area swept out per unit time) of average trajectories for different submovement speeds, calculated at the time of peak cursor speed.

(I–K) Equivalent analysis of a representative session with monkey R comprising 150 trials with 823 submovements.

(L) Average 2D LFP-PC trajectories for submovements, binned and color coded according to the direction of cursor movement. Arrows in the inset indicate the central direction of movement for each bin.

(M) Average LFP-PC trajectories in the plane of PC2 and PC3. The trajectories revolve around slightly different angular velocity vectors, indicated by arrows.

(N) Angular coefficient of determination for submovement direction, decoded from the orientation of LFP-PC angular velocity. Leave-one-out cross-validation was performed over every submovement in each data set. The plot shows the average CoD for validation submovements in each speed group, based on data from 13 sessions in three monkeys. Also shown is the average 95% percentile from shuffled data.

See also Movies S2 and S3.

single trials (Figure 1D, bottom). Coherence analysis over the whole session confirmed strong correlation between LFP-PCs and cursor speed at the 3 Hz frequency of submovements (Figure 2D).

The relationship between LFP oscillations and submovements was visualized by projecting the LFP trajectory over time onto the PC plane (Movie S1 available online). Due to the 90° phase difference between components, this trajectory was cyclic with constant direction of rotation. Each submovement was associated with a single LFP cycle, and the peak cursor speed occurred at a similar phase within each cycle (Figure 2E).

Submovement Kinematics Can Be Decoded from Areal Velocity of LFP Trajectories

To examine how LFP-PC trajectories were related to submovement kinematics, we binned submovements into nine equal groups according to peak cursor speed. Average LFP-PC trajectories for each group (Figures 2F and 2I; Movie S2) had constant direction and frequency of rotation (Figures 2G and 2J) but an areal velocity (area swept out per unit time about the origin; see Equation 2 in Experimental Procedures) that increased with cursor speed (Figures 2H and 2K). When binned according to the direction of submovements, average LFP trajectories in the PC plane appeared similar (Figure 2L). However, plotting the trajectories in 3D PC space revealed a subtle variation in the axis of rotation for different submovement directions (Figure 2M; Movie S3).

In three dimensions, areal velocity is conveniently represented by a vector aligned to the axis of rotation. We hypothesized that its magnitude should encode information about submovement speed, whereas its orientation might be informative of submovement direction. We tested this directly by decoding speed (or direction) from the magnitude (or orientation) of areal velocity vectors associated with individual submovements (see Experimental Procedures). Decoding performance for 13 data sets across three monkeys is summarized in Table S1, using a coefficient of determination (CoD) between zero (chance decoding) and one (perfect decoding). In every case, we obtained significant decoding of the speed from areal velocity magnitude, with mean (±SD) CoD = 0.30 ± 0.13. Decoding submovement direction from areal velocity orientation was significant in 12/13 sessions, with mean CoD = 0.15 ± 0.07. As might be expected, direction decoding was better for faster submovements (Figure 2N), with a CoD of 0.26 ± 0.17 for the fastest submovements. Although statistically significant, this nevertheless corresponds to an average decoding error of approximately 75°, only slightly better than chance (90° average decoding error).

In summary, the rhythmic structure of submovements is reflected in low-frequency M1 LFPs and can be revealed using PCA to reduce the dimensionality of the multichannel data. In the space defined by the first three PCs, each submovement is associated with a cyclic LFP trajectory, and the peak of the submovement occurs at a consistent phase of the cycle. The areal velocity of the trajectory is proportional to the speed of the submovement, whereas the axis of rotation provides statistically significant (albeit modest) information about the direction of movement.

Low-Frequency LFP Oscillations during Ketamine Sedation

On separate days, we recorded from the same electrodes during ketamine sedation (Figure 3A). M1 LFPs exhibited typical signatures of slow-wave sleep including spindles and large K complex potentials, thought to reflect transitions between cortical down and up states. Consistent with this, most neurons had low firing rates prior to each K complex, and fired particularly strongly during its rising phase and peak. Each K complex was associated with large-amplitude delta activity in the low-pass-filtered, mean-subtracted LFP, comprising either a single cycle or an extended burst of two or more cycles of low-frequency oscillation.

Phase of LFP Oscillations Relative to Submovements and K Complexes

Figure 4 compares cortical activity aligned to the peak speed of submovements (during task performance) and aligned to the peak of K complexes (under ketamine sedation). Both events were associated with phasic bursts of neural activity (Figure 4B) and phase-locked low-frequency LFP oscillations in M1 (Figure 4C), which were an order of magnitude larger in the sedated state. Note that the same color scheme is used throughout Figures 1 and 4C to represent the LFP phase *relative to submovements during task performance* but each electrode shows a similar phase *relative to the K complex under sedation*. As a result, average LFP-PC trajectories followed similar rotational cycles aligned to both submovements and K complexes (Figure 4D). We calculated the circular-circular correlation coefficient (ρ_{CC}) across electrodes of phase relative to submovements (during task performance) against phase relative to K complexes (under sedation). For the sample pair of movement and sedation sessions shown in Figure 4C, these phases were highly correlated ($n = 10$, $\rho_{CC} = 0.81$, $p = 0.025$; Figure 4E), and across all the data sets this correlation was significant ($p < 0.05$) in 11/13 pairs of sessions in three monkeys (mean ± SD; $\rho_{CC} = 0.75 \pm 0.25$; Table S1). Pooling all the sessions for each animal also yielded significant correlation (monkey D: $n = 52$, $\rho_{CC} = 0.63$, $p = 2 \times 10^{-5}$; monkey R: $n = 36$, $\rho_{CC} = 0.49$, $p = 0.002$; monkey S: $n = 45$, $\rho_{CC} = 0.81$, $p = 4 \times 10^{-6}$; Figure 4F). Moreover, advancing several microwires from the most superficial depth down through the gray matter revealed that both submovement- and K complex-related potentials underwent polarity reversals at the same depth, indicating a common cortical source (Figure S1).

In summary, the same patterns of cortical activity seen during isometric movements (and related to submovement kinematics) also arose endogenously in the absence of behavior, suggesting that intrinsic circuits rather than extrinsic sensorimotor feedback loops impose this dynamical structure on low-frequency cortical activity. Because isometric torque tracking and ketamine sedation are somewhat unnatural experimental conditions, we proceeded to examine whether the same low-frequency dynamics were also present in brain activity during more naturalistic conditions including sleep and unrestrained reach-to-grasp.

Low-Frequency LFP Oscillations during Natural Sleep and Free Reaching Movements

Two of our subjects often fell asleep at the end of recording sessions, providing an opportunity to examine low-frequency activity during natural sleep. As the eyes closed, large-amplitude slow waves were observed in the LFP (Figure 3B), of a comparable amplitude to sedation recordings and approximately an order of magnitude greater than that seen in the awake state. However, cortical activity appeared disorganized and lacked clear up-/downstate transitions or K complexes, which is consistent with stage 1 sleep.

In addition, we collected data when the same animals retrieved food from wells in a Klüver board with the arm unrestrained. In general, LFPs showed less rhythmicity than during isometric tracking, but there were nevertheless periods of pronounced low-frequency oscillation in M1 with a phase that varied systematically across electrodes (Figure 3C). We did not

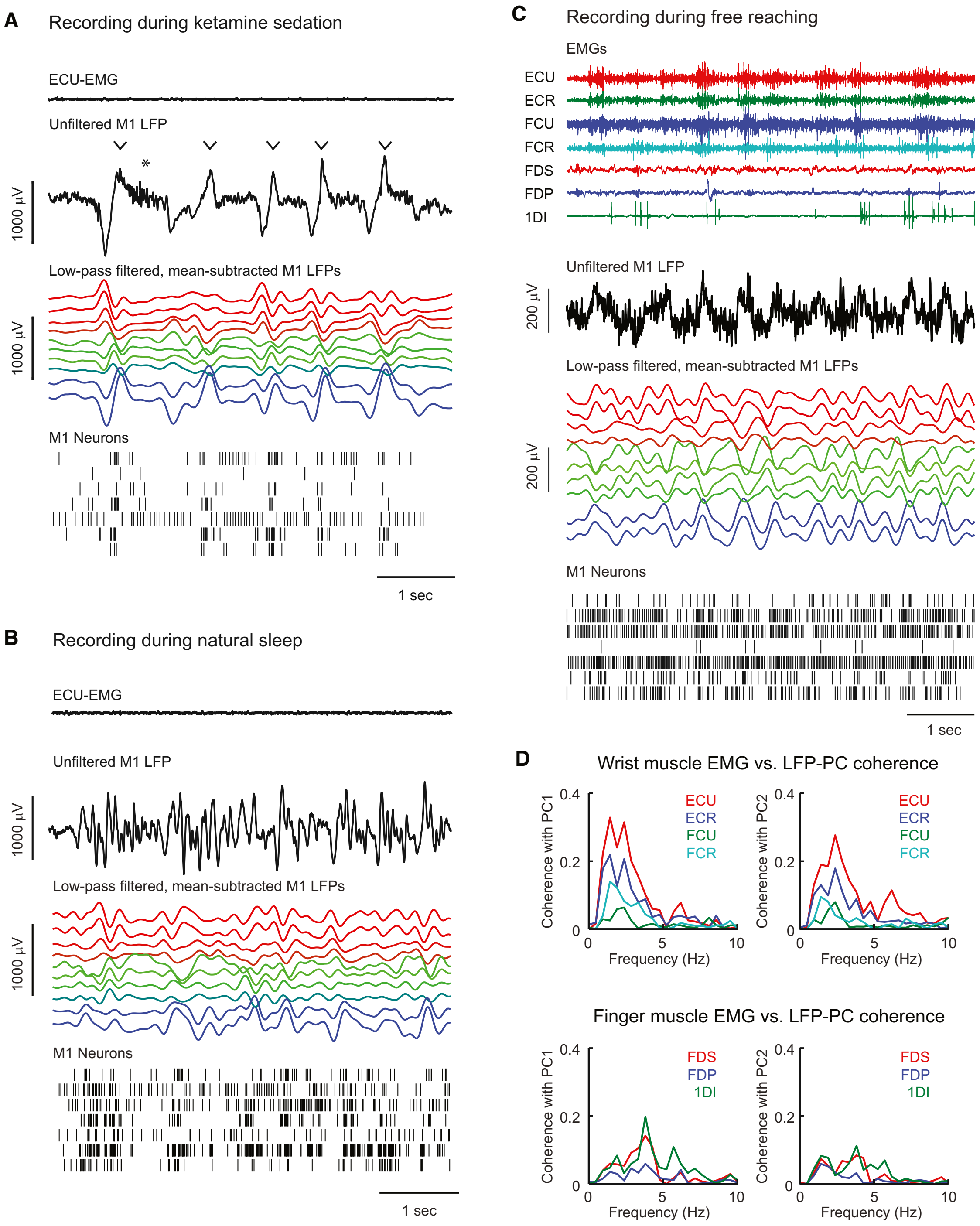

Figure 3. Low-Frequency Cortical Dynamics during Ketamine Sedation, Natural Sleep, and Free Reaching

(A) EMG, unfiltered LFP, processed LFP, and spike rasters during ketamine sedation following the session in Figure 1. Arrowheads indicate K complexes, sometimes associated with spindles (*). LFP traces are ordered and color coded as in Figure 1.

(B) Equivalent recordings during natural sleep at the end of the session in Figure 1.

(C) Equivalent recordings during retrieval of food from small wells in a Klüver board. In addition to the wrist muscles named in Figure 2, EMG was recorded from flexor digitorum profundus (FDP), flexor digitorum superficialis (FDS), and first dorsal interosseous (1DI), which act on the fingers.

(D) Coherence spectra between M1 LFP-PCs and wrist and finger muscles during free reaching exhibit low-frequency coherence peaks.

measure kinematics during these complex whole-limb movements, but characterized behavior instead using EMGs recorded from multiple hand and wrist muscles. When projected onto the PC plane determined from isometric task recordings, the first two LFP-PCs were coherent with rectified EMG in the delta band (Figure 3D), suggesting a consistent relationship between LFP and muscle activity even during unrestrained reach-to-grasp.

Common Low-Frequency LFP Dynamics across Behavioral States

Figure 5A shows power spectra for a representative M1 LFP under the four behavioral states: isometric task performance, free reaching, natural sleep, and ketamine sedation. It is clear that the spectra vary substantially across conditions. Awake isometric and naturalistic reaching behaviors are characterized by a peak in the beta band around 20 Hz, whereas sleep and sedation recordings show increased power at low frequencies. A clear peak in the delta band is seen during sedation (but not sleep), whereas awake behaviors are associated with a broad distribution of power at low frequencies.

The event-triggered analysis used in Figure 4 was not applicable to these naturalistic recordings because the sleep data lacked K complexes, and the timing of submovements could not be accurately determined for free movements. Therefore, we examined whether a conserved dynamical structure could be found in the correlation structure between multichannel LFPs, and between LFPs and spiking activity, under the four different behavioral states. In all analyses, we used the same 2D projection of the LFP data, which was determined by PCA of the low-pass-filtered isometric torque data (LFP-PCs).

Despite differences in LFP power spectra, the low-frequency correlation structure between LFP-PCs was preserved under all four behavioral states (Figure 5B). Cross-correlation of both unfiltered and low-pass-filtered LFP-PCs revealed strong, consistent peaks and troughs separated by about 150 ms, corresponding to an oscillatory cycle of around 3 Hz. This is not a trivial consequence of PCA decomposition, because although PCs must be uncorrelated at zero lag, there is no reason why they should be strongly correlated at any other lag. Moreover, it is not trivial that LFPs recorded under other behavioral states, when

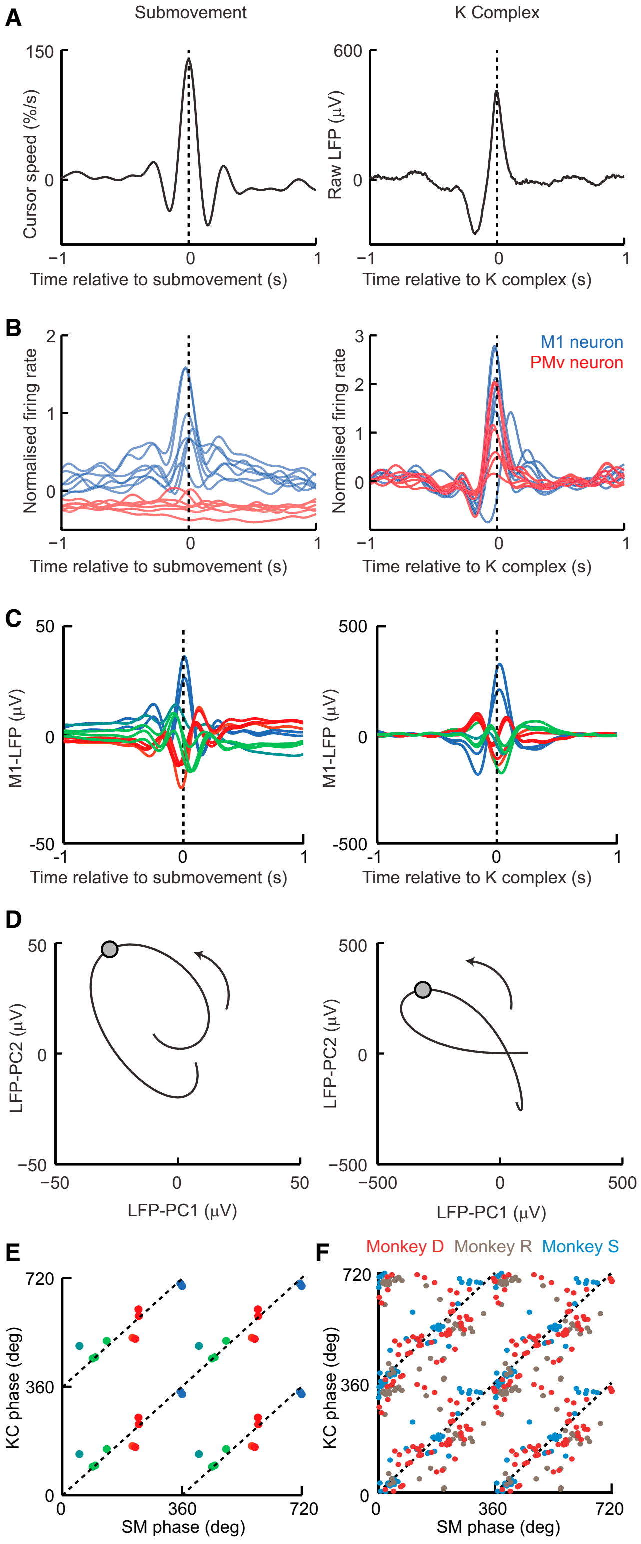

Figure 4. Submovement- and K Complex-Related Activity Share a Common Low-Frequency Phase Structure

(A) Left: average cursor speed aligned to a peak speed of 2,063 submovements. Right: average surface-referenced unfiltered LFP aligned to the peak of 197 K complexes. Data are from monkey D, same sessions as in Figures 1 and 3.

(B) Average normalized firing rate of seven neurons in M1 (blue) and six neurons in PMv (red) relative to submovement (left) and K complex (right).

(C) Average low-pass-filtered, mean-subtracted LFP from ten M1 electrodes relative to submovement (left) and K complex (right). Traces in both plots are color coded according to phase relative to submovements.

(D) Average submovement-triggered (left) and K complex-triggered (right) LFP-PC trajectories, plotted over 200 ms on either side of the trigger event (indicated by circles). All data are projected onto the PC axes determined from LFPs recorded during isometric task performance.

(E) LFP phase relative to submovement (SM phase) plotted against LFP phase relative to K complex (KC phase) for each M1 electrode (unwrapped over two full cycles). Dashed lines indicate equality. Points are color coded according to LFP phase relative to submovements.

(F) SM phase plotted against KC phase for all LFP recordings over 13 sessions in three monkeys.

Data are presented in Table S2. See also Figure S1.

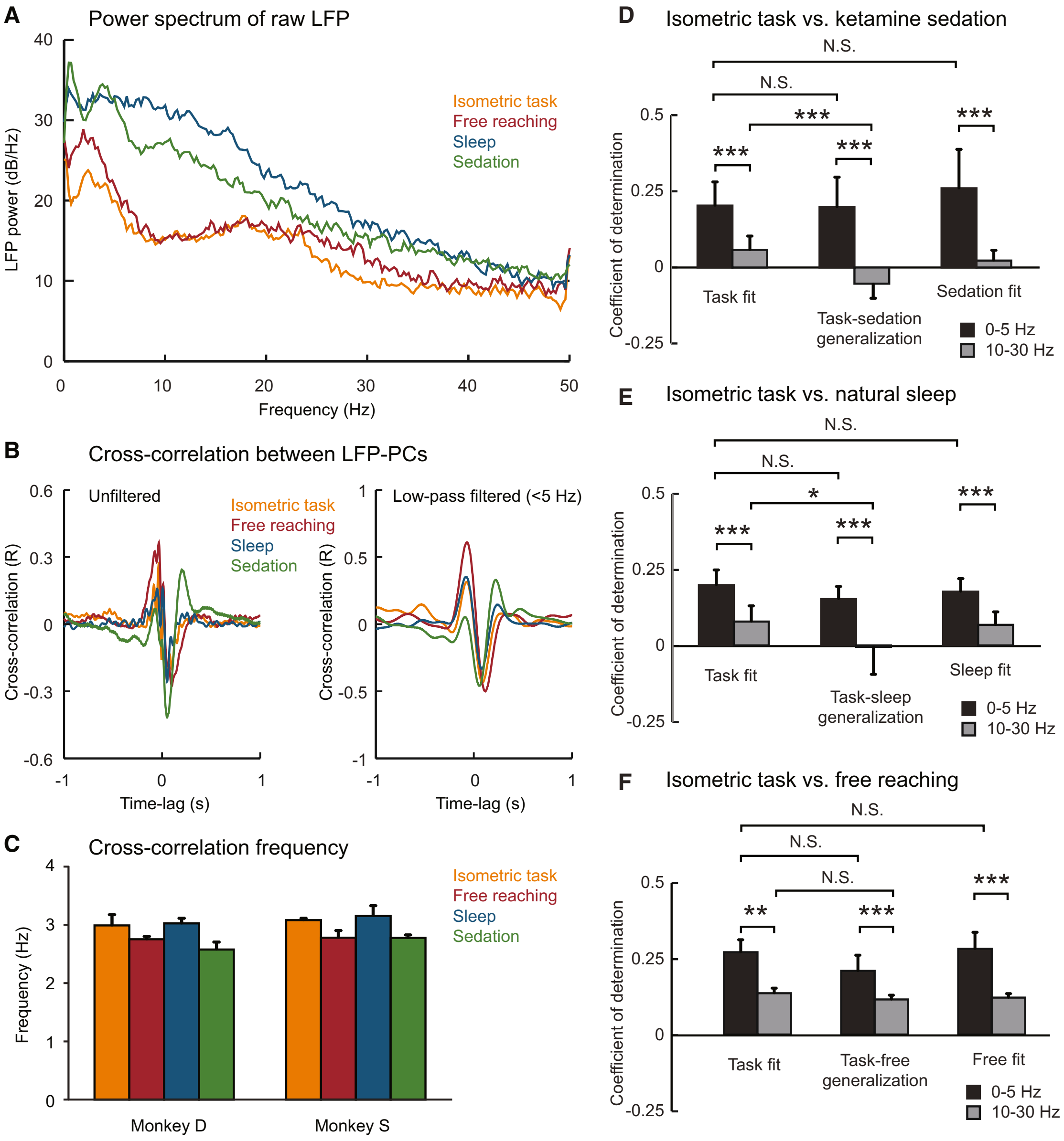

Figure 5. Consistent Low-Frequency LFP Dynamics across Behavioral Conditions

(A) Power spectrum of unfiltered M1 LFPs during isometric task performance, free reaching, natural sleep, and ketamine sedation.

(B) Cross-correlation (normalized R values for each time lag) between unfiltered (left) and low-pass-filtered (right) LFP-PCs under four behavioral states. All data are projected onto the PC axes determined from low-frequency LFPs recorded during isometric task performance.

(C) Average frequency of LFP-PC correlation (determined from the time interval between cross-correlation peak and trough) for monkeys D and S. In both animals a small but consistent reduction in frequency is observed during free reaching and ketamine sedation.

(D) Coefficient of determination for a linear dynamical model fit to delta band (black) and beta band (gray). The plot compares the quality of the model fitted on isometric task data and tested on the same task data (task fit), the model fitted on task data and tested on sedation data (task-sedation generalization), and the model fitted to sedation data and tested on sedation data (sedation fit). Thirteen session pairs in three animals; data are presented in Table S3.

(E) Equivalent plot showing the model fitted on isometric task data generalizes to natural sleep. Nine session pairs in two animals; data are presented in Table S4.

(F) Equivalent plot showing the model fitted on isometric task data generalizes to free reaching. Ten session pairs in two animals; data are presented in Table S5.

Error bars indicate SD. N.S., not significant, $p > 0.05$; *$p < 0.05$; **$p < 0.01$; ***$p < 0.001$; paired t test.

projected onto the PC axes determined from the isometric data, should exhibit *the same* correlation structure. Indeed, this analysis revealed a subtle but systematic difference in the frequency of oscillation (determined from the interval between cross-correlation peak and trough). In both animals for which we recorded in all four conditions, the frequency of correlation was highest (~3 Hz) during isometric movements and natural sleep, and slightly lower (~2.8 Hz) during free reaching and ketamine

sedation (Figure 5C). Although small, this difference was individually significant in both animals (one-factor ANOVA; monkey D: $F_{3,16} = 11.4$, $p = 3 \times 10^{-4}$; monkey S: $F_{3,10} = 8.0$, $p = 0.005$).

Another way to visualize the similarity in correlation structure is to plot the LFP trajectory over time in the PC plane. Movie S4 shows real-time LFP data recorded in the different conditions, alongside its PC projection (note that the awake data are expanded 2-fold to compensate for the increase in slow-wave amplitude during sleep and sedation). In all cases the LFP trajectory rotated in the same direction, with a frequency of around 3 Hz. We quantified the extent to which these LFP trajectories could be captured by a single first-order linear dynamical equation of the form

$$\dot{\boldsymbol{x}}(t) = \boldsymbol{A}.\boldsymbol{x}(t), \tag{1}$$

where the time evolution of the first two LFP-PCs, $\boldsymbol{x}(t)$, is determined only by a 2 × 2 matrix, $\boldsymbol{A}$, with a trace equal to zero. Figure S2A shows this procedure applied to LFPs recorded during the isometric task. Solutions of Equation 1 are closed elliptical trajectories with constant frequency and direction of rotation (Figure S2C), similar to the real data (Figure S2D). The three free parameters of $\boldsymbol{A}$ (the fourth is fixed by the trace constraint) effectively determine the frequency, orientation, and eccentricity of trajectories. Therefore, the extent to which a single matrix $\boldsymbol{A}$ can describe the time evolution of LFP-PC trajectories provides a measure of the consistency of the underlying dynamics.

We quantified the fit over sessions of isometric task performance using the coefficient of determination (Equation 10 in Experimental Procedures) and obtained an average (±SD) CoD of 0.20 ± 0.08 (n = 13 sessions in three animals; Table S3). The quality of this fit is not a trivial consequence of the orthogonality of PCs, because most orthogonal signals cannot be described by Equation 1. When the same analysis steps (low-pass filtering, mean referencing, PCA, and model fitting) were applied to equivalent lengths of white noise, the 95% percentile of the distribution of the resultant CoD was only 0.0013. Moreover, not all oscillatory activity can be described by Equation 1. Equivalent analysis of beta-band LFP data (filtered between 10 and 30 Hz; Figure S2B) yielded an average CoD of only 0.06 ± 0.05, significantly worse than the low-frequency fit (n = 13, t = 6.8, $p = 2 \times 10^{-5}$, paired t test; Figure 5D). This was not due to an absence of signal at this frequency, because beta-frequency oscillation was evident in the raw signal (Figure S2A) and power spectrum (Figure 5A). Rather, the oscillation at this frequency comprised predominantly a single phase leading to a high proportion of variance in the first PC (Figure S2E), whereas the second PC had no consistent phase relationship. Therefore, trajectories in the PC plane lacked rotational structure (Figure S2D), and hence could not be described by first-order linear dynamics.

Next, we tested how well the model that best described the isometric task data could explain LFPs recorded under ketamine sedation. We applied the best-fit parameters obtained from the task recordings to predict the time derivative of the sedation data using Equation 1 and achieved a comparable CoD of 0.20 ± 0.10. This was significantly better than the generalization of the beta-band model, which failed to fit these frequencies in the sedation data (mean CoD = −0.05 ± 0.05, n = 13, t = 8.5, $p = 2 \times 10^{-6}$, paired t test). For comparison, the model with parameters best fit to the sedated state explained the delta-band data only marginally better, with a CoD of 0.26 ± 0.13 (Figure 5D; Table S3), whereas the best fit to the beta-band activity remained poor (CoD = 0.02 ± 0.03).

Similar results were obtained for the generalization of the isometric task model to data recorded during natural sleep (Figure 5E; Table S4) and free reaching (Figure 5F; Table S5). In both cases, the model parameters that best fit the task data captured a significantly higher proportion of LFP dynamics in the delta band compared with the beta band, and the quality of the fit was only marginally improved by fitting model parameters to the corresponding behavioral state.

These analyses confirm the consistent correlation structure in multichannel M1 LFP activity under all four behavioral states, albeit with a minor (~10%) reduction in frequency during free reaching and sedation. Next, we examined whether similar slow LFP oscillations were also observed in ventral premotor cortex (PMv), and how they related to neural activity in each area.

Distinct Low-Frequency LFP Oscillations in M1 and PMv during Task Performance

Figure 6 compares M1 and PMv activity during a single trial of isometric task performance, and Figures 7A–7C show average data for an entire session aligned to the end of each successful trial. Firing rates in M1 (Figures 6B and 7B) were highest during the rising torque phase, as the animal made multiple submovements to acquire peripheral targets. By contrast, PMv firing rates were highest after the end of the trial, as the animal took a food reward with the ipsilateral limb. This is consistent with greater bilateral tuning of premotor neurons (Hoshi and Tanji, 2006), as well as with a strong preference for object-grasping movements within the bank of the arcuate sulcus (Umilta et al., 2007).

These distinct periods of high neuronal activity were each associated with low-frequency LFP activity within the same cortical area (Figure 6C). In both M1 and PMv, the low-pass-filtered LFP could be decomposed into two orthogonal components (Figure 6D). Submovements during the trial were phase locked to the M1 cycle (Figure 6E), but had no consistent relationship to the PMv LFP. The areal velocity of the LFP-PC trajectory in M1 and PMv was maximal during periods of high neural activity in the same cortical area (Figures 6F and 7C). LFP-PCs within each area exhibited a similar low-frequency correlation structure (Figure 6G), indicating a consistent phase lag throughout the recording. However, the oscillations in each area were largely independent of each other during the isometric task, occurring at different phases of the task. As a result, correlations between LFP-PCs across areas were weaker than within areas (Figure 6H).

By contrast, neurons in M1 and PMv were coactive during free reaching-to-grasp with the contralateral limb (Figure S3). In this case, slow oscillations in both areas were phase locked, leading to robust correlation between LFP-PCs across areas. Finally, neural activity in both M1 and PMv under sedation was synchronized to K complexes (Figures 7D and 7E; Figure S4). Each K complex was also associated with synchronous bursts of low-frequency oscillation, resulting in peaks of LFP-PC areal velocity in both areas (Figure 7F).

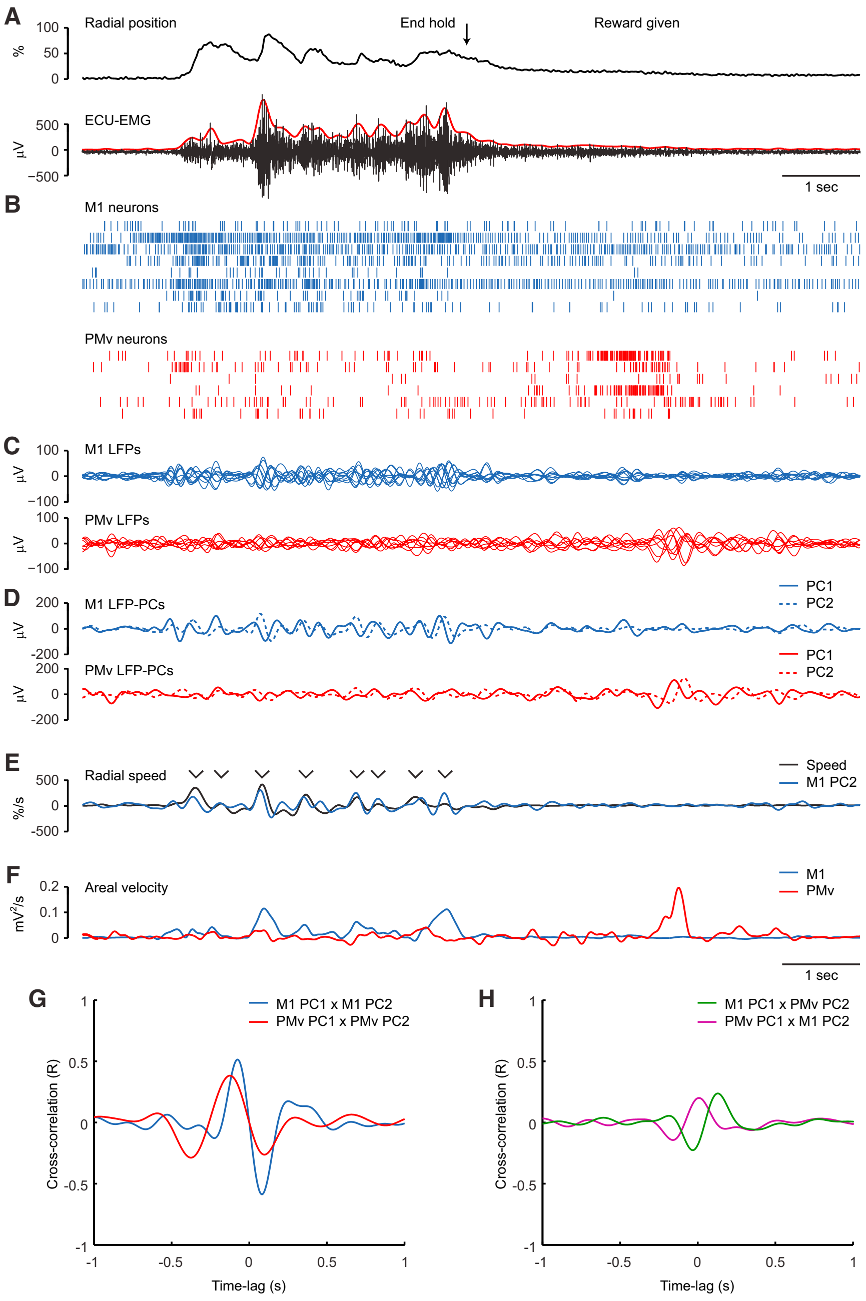

Figure 6. M1 and PMv Are Active during Distinct Phases of Isometric Task Performance

(A) Radial cursor position for a representative trial of the isometric task. After the peripheral target is acquired (End hold), the monkey takes a food reward with the hand ipsilateral to the recording sites. Also shown is raw (black) and rectified, smoothed (red; not to scale) EMG from a wrist extensor muscle, in which the submovement structure is more clearly evident.

(B) Spike rasters for eight neurons in M1 (blue) and six neurons in PMv (red). Note that M1 neurons fire with contralateral isometric wrist submovements, whereas PMv neurons are active as the monkey takes food with the ipsilateral limb.

(C) Low-pass-filtered, mean-subtracted LFPs recorded from ten electrodes in M1 and eight electrodes in PMv.

(D) LFP-PCs calculated from M1 and PMv recordings.

(E) Radial cursor speed and M1 LFP-PC2 overlaid. Arrowheads indicate identified submovements with a peak speed exceeding 30%/s. Note that submovements are phase locked to the M1 oscillation, although in this trial they do not occur on every cycle.

(F) Areal velocity in the PC plane for M1 and PMv LFPs. Increased areal velocity in M1 coincides with M1 neural activity, whereas increased areal velocity in PMv coincides with PMv neural activity.

(G) Cross-correlation between LFP-PCs within the same cortical area.

(H) Cross-correlation between LFP-PCs across cortical areas.

Data are from monkey D. See also Figures S3 and S4.

Neuronal Firing in M1 Is Phase Locked to Slow Oscillations during Movement, Sleep, and Sedation

Last, we examined how spiking activity was related to the phase of the low-frequency oscillation in each area. Figure 8A and Movie S5 show sample spike-triggered average trajectories of M1 and PMv LFP-PCs for the same set of neurons recorded during the four behavioral states. During isometric task performance, neurons showed greater locking to LFPs within the same cortical area, as expected from the dissociation of activity patterns during different task phases (Figures 7B and 7C). However, during free reaching, sleep, and sedation, spike activity in both M1 and PMv was associated with cyclical LFP-PC trajectories within and across areas, consistent with the synchronization of low-frequency rhythms under these conditions.

To assess phase locking to M1 LFP cycles across different data sets, we rotated the M1 LFP-PC plane such that the peak speed during isometric submovements occurred at a phase of zero. Across the population, M1 neurons were significantly phase locked to M1 LFP oscillations in all three animals (Figure 8B), with an average preferred phase that preceded peak speed, consistent with these neurons having a causal role in movement. During task performance, PMv neurons in two out of the three animals (monkeys D and R) did not show consistent locking to M1-LFP cycles, whereas in the third animal (monkey S) the distribution of preferred phases was significantly nonuniform but nevertheless broad relative to M1 neurons. However, during free reaching, natural sleep and ketamine sedation, neurons in both M1 and PMv became synchronized and fired at a similar preferred phase of the M1 cycle in all three animals.

In summary, the relationship between spiking activity and LFPs suggests that each cortical area is governed by its own

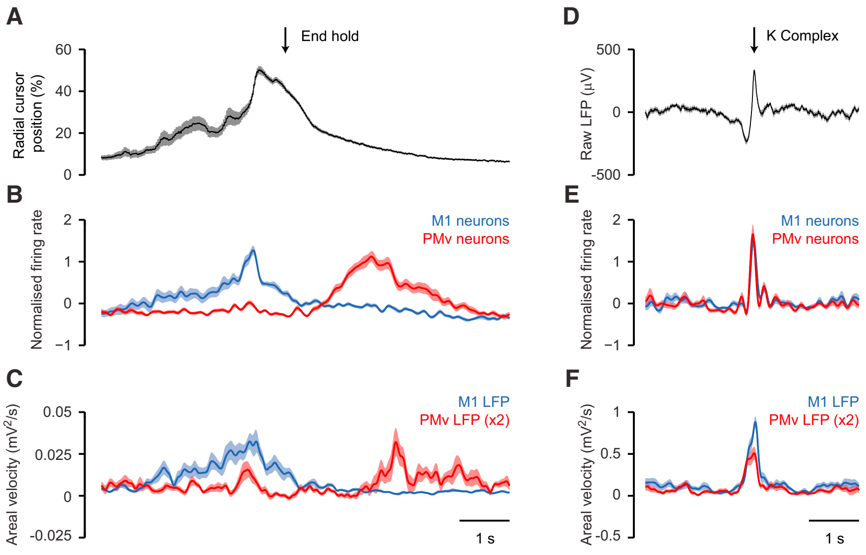

Figure 7. M1 and PMv Activity during Isometric Task Performance and Ketamine Sedation

(A) Radial cursor position aligned to the end of successful hold periods for peripheral targets, averaged across 40 trials from the session shown in Figure 6.

(B) Average normalized (to zero mean and unity standard deviation) firing rate for eight M1 neurons and six PMv neurons, aligned to the end of the hold period. M1 activity is highest as the monkey generates torque with the contralateral wrist to reach targets. PMv activity is highest after the successful trial, corresponding to taking food reward with the ipsilateral limb.

(C) Average areal velocity in the PC plane of M1 and PMv LFPs, aligned to the end of the hold period. The profile of areal velocity during task performance mirrors the dissociation seen in neural activity across areas. Note that the vertical scale for PMv areal velocity is expanded ×2 for ease of comparison.

(D) Average unfiltered, surface-referenced M1 LFP aligned to the peak of 48 K complexes during ketamine sedation.

(E) Average normalized firing rate for the same M1 and PMv neurons, aligned to K complexes. Neural activity in both M1 and PMv is maximal during the rising phase and peak of the K complexes.

(F) Average areal velocity in the PC plane of M1 and PMv LFPs, aligned to K complexes.

Shading indicates SEM across trials or K complexes.

intrinsic dynamics, allowing distinct slow oscillations to emerge in M1 and PMv when those areas are individually active during different phases of the isometric task. However, during free behavior involving coactivation of M1 and PMv, as well as during sleep and sedation, the slow oscillations become coupled across cortical areas.

DISCUSSION

A Common Structure Underlies Low-Frequency Motor Cortex Activity during Movement and Sedation

We have described a common 3 Hz correlation structure in LFP recordings during an isometric movement task, free reaching, natural sleep, and ketamine sedation. Individual LFPs exhibited oscillatory activity, albeit of lower amplitude in the awake state, with a consistent distribution of phase across electrodes relative to submovements and K complexes. Because this phase distribution was preserved across behavioral states, the multielectrode LFP could be decomposed into two orthogonal components that evolved according to the same underlying dynamics during all behavioral conditions. During isometric task performance, M1 neurons fired at a consistent phase of the cortical cycle, and this modulation of descending drive led to a 3 Hz submovement structure in muscle activity and movement kinematics. A similar cycle was evident during free reaching movements, which also comprise submovements (Milner and Ijaz, 1990; Roitman et al., 2004), although peripheral interactions with limb biomechanics and afferent feedback may lower the frequency and obscure the clear rhythmicity seen in isometric tasks. Interestingly, the isometric task also revealed a dissociation of neural activity within M1 and PMv during different task phases, each associated with a distinct low-frequency oscillation. However, during sleep and under sedation, these rhythms became coupled across different areas, which may explain the increased slow-wave amplitude seen in these brain states.

Functional Role of Slow Oscillations

It has previously been thought that the frequency of submovements during visuomotor tracking was determined by feedback loop delays, because their rhythmicity is disrupted under conditions of absent or delayed visual feedback (Miall et al., 1986, 1993; McAuley et al., 1999; Miall and Jackson, 2006). However, our finding of a common oscillatory structure in the cortical LFP that is (1) coherent with movement speed and (2) present during sleep and sedation reveals an intrinsic periodicity in motor circuitry at the submovement frequency. Submovement durations are relatively unaffected by movement speed (Miall et al., 1986; Roitman et al., 2004; Pasalar et al., 2005; Selen et al., 2006), target size (Selen et al., 2006), arm stiffness (Selen et al., 2006), or learning novel visuomotor mappings (Sailer et al., 2005). One possibility is that the intrinsic dynamics are tuned appropriately for visuomotor control such that different phases of the cycle are associated with the various computations involved in planning and generating the next submovement based on feedback from the previous one. In fact, adaptation to delayed visual feedback is extremely limited (Miall and Jackson, 2006), suggesting that the motor system may in fact be tuned only to a narrow range of naturally occurring loop delays. Indeed, Kunesch et al. (1989) concluded that the temporal characteristics of manipulative hand movements requiring tactile feedback were determined not by (shorter) sensorimotor loop delays but instead by central neural mechanisms responsible for interpreting sensory inputs. This would be consistent with a common intrinsic oscillator shaping the structure of feedback-controlled movements, irrespective of the feedback modality. Finally, it is interesting to note that during verbal articulation there

is coherence between cortical signals and mouth EMG at a frequency of 2–3 Hz, which reflects the spontaneous rhythmicity of speech (Ruspantini et al., 2012).

The Origin of Low-Frequency Cortical Dynamics

Care must be taken when inferring neural substrates of LFP activity, because synaptic and intrinsic currents from multiple neuronal populations contribute to the extracellular field (Buzsáki et al., 2012). Moreover, rotation in the PC plane does not require underlying oscillatory sources that are orthogonal, because any consistent phase difference, or a traveling wave appearing with a different phase on each electrode, could equally be decomposed into orthogonal components (Rubino et al., 2006; Murphy et al., 2009; Nauhaus et al., 2009; Ray and Maunsell, 2011).

Importantly, the distribution of preferred phase for neural firing was narrow compared to the LFP (Figure S5A). Moreover, during sleep and sedation, this phase was common to neurons in both M1 and PMv (Figure 8B). This appears incompatible with a traveling wave, which would cause neurons at different locations to fire at different preferred phases of the global cycle. Churchland et al. (2012) reported complex, multiphasic patterns of cortical activity that could be projected onto a plane using the jPCA method to reveal consistent cycles with notable similarity to the LFP trajectories we describe here. However, it is not clear from that study whether all phases of the cycle were represented equally, because the jPCA method is again based on orthogonal projections of the neural activity. Consistent with our observations, Riehle et al. (2013) found that movement-related potentials were composed of multiple components with amplitude and latency that varied systematically across the cortical surface, even though recorded neurons tended to be maximally active around movement onset.

A parsimonious explanation of the consistent correlation structure we describe is that the multichannel LFP comprises a mixture of at least two underlying sources with a fixed time/phase delay (Figures S5B and S5C). If one source reflects (relatively) synchronous neural activity occurring around submovements and K complexes, what then is the source of the second component? One possibility is that neural activity at other phases is undersampled in our recordings, either because the neurons are located in a different area of cortex or a subcortical structure or have smaller soma size (for example, inhibitory interneurons). An alternative explanation is that the field potential associated with synchronous neural activity may be composed of multiple sources with different time courses. These sources are cortical, because submovement- and K complex-related LFP oscillations underwent polarity reversal within the gray matter, and we speculate that they may reflect excitatory and inhibitory synaptic potentials contributing to the generation of low-frequency rhythms. Delta oscillations can arise in the thalamus due to low-threshold calcium currents active in the hyperpolarized state (Amzica et al., 1992; Destexhe and Sejnowski, 2003). However, in the awake state, thalamic neurons are depolarized and generally fire in a tonic mode (Steriade and Llinás, 1988), suggesting that the low-frequency dynamics we observe during behavior may relate to a cortical delta rhythm that has recently been characterized in slice preparations. This rhythm originates from intrinsic bursting cells in layer V that activate a source of $GABA_B$-mediated inhibition (Carracedo et al., 2013). The slow kinetics of this G protein-coupled receptor lead to sustained hyperpolarizing currents that can be delayed by several hundred milliseconds relative to inhibitory cell activity. These slow currents are observed in the LFP (Dine et al., 2014), and might be expected to contribute a low-frequency component with a substantial phase lag relative to ionotropic currents. Nevertheless, occasional bursting has been reported in the thalamus in the awake state (Guido and Weyand, 1995), and the relative contributions of cortical and corticothalamic mechanisms in generating delta activity in vivo during behavior and sleep remain an important area for further investigation.

Kinematic Information in LFP Trajectories

Low-frequency LFPs have several practical advantages for BMIs (Rickert et al., 2005; Bansal et al., 2011; Hwang and Andersen, 2013), but our understanding of how these signals arise and how best to extract information from them is limited. We found that the areal velocity swept out by LFP trajectories was proportional to movement speed, and suggest that this may prove a useful feature to examine for BMI applications, as it is robust to sources of synchronous noise (because correlated signals lead to radial trajectories). In 3D PC space, there was a slight variation in the axis of rotation for different directions of movement. In effect, the first two PCs captured the LFP trajectory that was common across all submovements, whereas the third component reflected more subtle variation in the neuronal sources associated with different directions (Waldert et al., 2009). These observations suggest that understanding the lawful dynamics that generate low-frequency behaviors may inform and constrain the search for more sophisticated approaches to decoding kinematics from LFPs.

Conclusions

By examining the dynamics of motor cortex activity, we can unite two previously distinct phenomena: the rhythmicity of submovements during isometric tracking and delta oscillations during sleep and under sedation. In both cases, cortical neurons fire at distinct phases of the same underlying 3 Hz LFP cycle, and thereby impose this frequency on behavior via modulation of the descending drive to muscles. We suggest that this intrinsic rhythmicity reflects an underlying organization of motor cortical circuits engaged in feedback control of movement.

EXPERIMENTAL PROCEDURES

Isometric Movement Task

Experiments were approved by the local ethics committee and performed under appropriate UK Home Office licenses in accordance with the Animals (Scientific Procedures) Act 1986. Three purpose-bred female rhesus macaques (monkey R: 5 years old, 5 kg; monkey D: 6 years old, 6.5 kg; monkey S: 5 years old, 5.4 kg) were trained to control a cursor by generating isometric flexion-extension (vertical) and radial-ulnar (horizontal) torque with the left wrist restrained in pronated posture to move to eight peripheral targets presented in a pseudorandomized center-out sequence on a computer monitor. Wrist torque was measured using a six-axis force/torque transducer (Nano25; ATI Industrial Automation). Cursor position was expressed as percentage of the distance to screen edge, with 100% corresponding to a torque of 0.67 Nm. Targets were centered at 70% of the distance to screen edge and had a diameter of 25%.

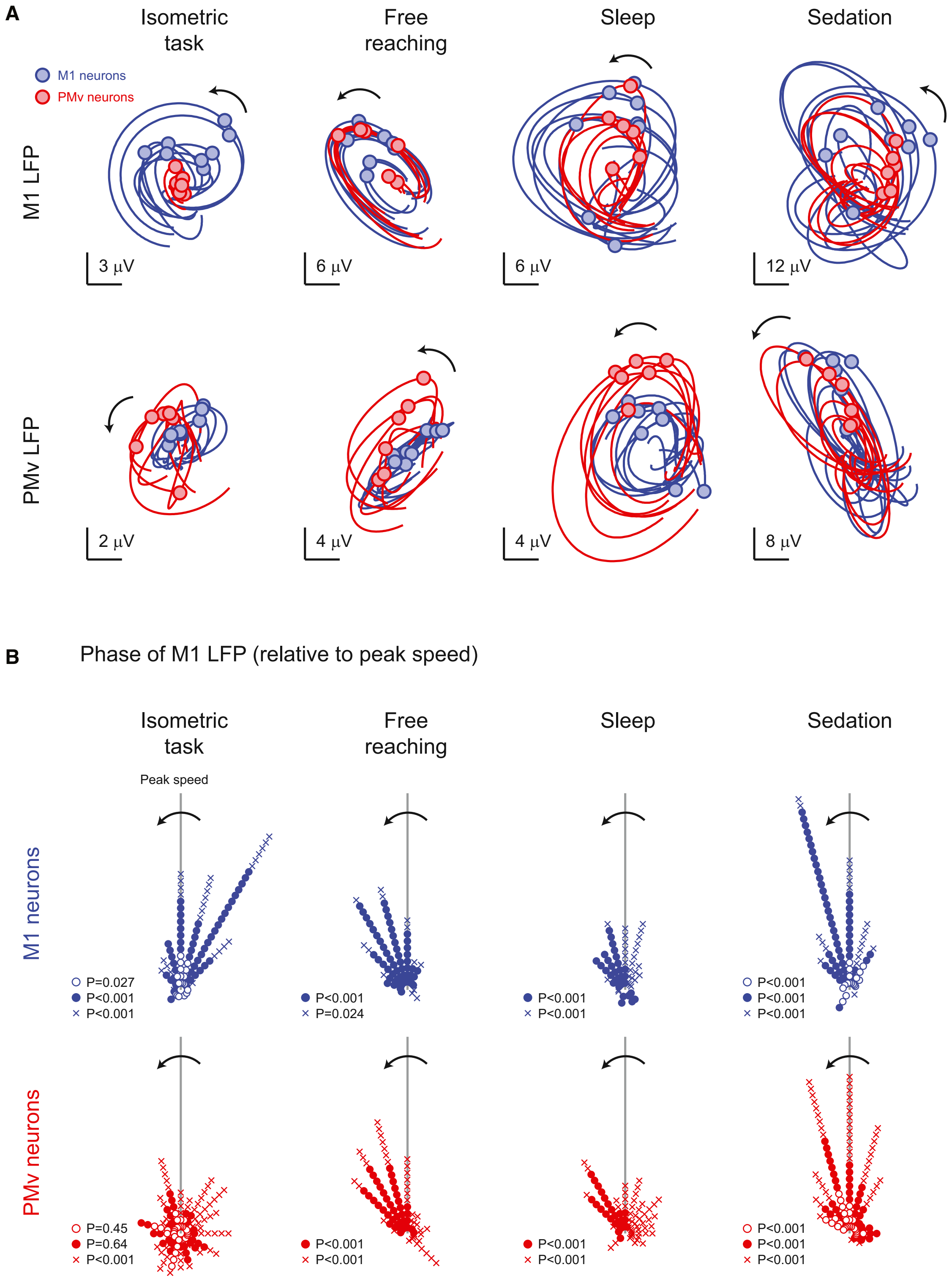

Figure 8. Phase Locking of Neural Activity to the Cortical Cycle during Isometric Movement, Free Reaching, Natural Sleep, and Ketamine Sedation

(A) Spike-triggered average LFP-PC trajectories for eight M1 neurons (blue) and six PMv neurons (red) over 200 ms before and after spike time (indicated by circles). Top row: averages of M1 LFPs; bottom row: averages of PMv LFPs. In all cases, the data are projected onto PC axes

(legend continued on next page)

Surgical Procedures

After training, we implanted EMG electrodes onto forearm and hand muscles, tunneled subcutaneously to a connector on the head. In a separate surgery, two custom arrays of 12 moveable 50 μm diameter tungsten microwires (impedance ~200 kΩ at 1 kHz) were implanted into the right M1 and PMv (Jackson and Fetz, 2007). All surgeries were performed under sevoflurane anesthesia with postoperative analgesics and antibiotics.

Electrophysiological Recording

Head-free recordings were made using unity-gain headstages followed by wide-band amplification and sampling at 24.4 ksp/s (sp, sample) (System 3; Tucker-Davis Technologies). LFPs were digitally low-pass filtered at 300 Hz and recorded at 488 sp/s. EMGs were amplified (×1,000) and band-pass filtered between 10 and 5,000 Hz (model 1700; AM Systems) before sampling at 12.2 ksp/s.

Data Set

Kinematic decoding was performed on 13 sessions (monkey R: 4; monkey D: 6; monkey S: 3). Sedation data (at least 5 min per session) were recorded on separate days after induction with ketamine (10 mg/kg, intramuscularly; i.m.) and medetomidine (0.02 mg/kg, i.m.). We report 13 pairs of movement and sedation sessions (separated by no more than 3 days) for all animals (monkey D: 5; monkey R: 4; monkey S: 4). In monkeys D and S, we collected natural sleep data at the end of behavioral sessions, and report four and five pairs of sessions, respectively. In monkeys D and S, we also collected data while animals retrieved food from a Klüver board, and report six and four such sessions. On average, 15 neurons were recorded per session.

Data Preprocessing

Offline analyses were performed in MATLAB (MathWorks). LFPs were visually inspected and electrodes with excessive mains noise or artifacts were discarded. Remaining LFPs recorded during isometric task performance were separated by area (M1 and PMv) and processed by low-pass filtering (5 Hz, four-pole, zero-phase digital Butterworth filter), mean referencing (i.e., subtraction of the mean LFP across electrodes within the same cortical area), and dimensionality reduction using standard PCA. The PC plane was oriented such that the predominant rotational structure during task performance was in the anticlockwise direction. In all analyses, LFPs recorded during other behavioral states (free reaching, sleep, and sedation) were always projected into the same PC space obtained from the corresponding isometric task data set. We refer to these projections throughout as LFP-PCs.

Cursor speed was calculated as the derivative of the magnitude of the 2D torque vector, that is, the radial component of velocity with a positive sign for movements away from the center of the screen. Submovements were defined by a peak speed exceeding 30%/s. K complexes were identified from a single surface-referenced LFP channel as the peak of a positive deflection that exceeded 250 μV.

Coherence spectra were calculated between unfiltered cursor position and speed; rectified EMG and LFP-PCs used a 2,048-point rectangular window with no overlap. Although the PC axes were determined from low-pass-filtered data, we used unfiltered LFP projections for coherence spectra so as to include frequencies above the filter cutoff.

Online, semisupervised spike classification used principal component feature extraction and K means clustering (SpikePac; Tucker-Davis Technologies). Firing-rate profiles for each neuron were calculated offline by binning spike events (into 488 bins/s), low-pass filtering at 5 Hz, and normalizing to zero mean and unity standard deviation across the entire recording.

Areal Velocity of LFP-PC Trajectories

Submovements were binned into nine groups of equal size according to increasing peak cursor speed, or alternatively into six groups according to submovement direction. 2D LFP-PC trajectories from 200 ms before to 200 ms after the midpoint of each submovement were averaged within each group and quantified using areal velocity (ν_{areal}) and frequency of rotation (f):

$$\boldsymbol{v}_{areal}(t) = \frac{1}{2}\boldsymbol{x}(t) \times \dot{\boldsymbol{x}}(t) \tag{2}$$

$$|\boldsymbol{v}_{areal}(t)| = \frac{1}{2}|\boldsymbol{x}(t)|^2 .2\pi .f(t). \tag{3}$$

Here, $\boldsymbol{x}(t)$ is a 2D or 3D vector of LFP-PCs at time t, $\dot{\boldsymbol{x}}(t)$ is its derivative with respect to time, and × denotes the vector cross-product. For each submovement group, rotation frequency and areal velocity were measured at the time of peak cursor speed.

Areal Velocity Decoding of Single-Submovement Kinematics

We used the average 3D areal velocity vector (Equation 2) from 200 ms before to 200 ms after the time of peak speed to decode the kinematics of individual submovements with leave-one-out cross-validation, as follows.

(1) We parameterized the relationship between areal velocity $\boldsymbol{v}_i$ (for submovement i) and the speed and direction of that submovement (s_i, θ_i) assuming (1) for a given direction of submovement, the areal velocity magnitude increased linearly with cursor speed, and (2) for a given speed of submovement, the orientation of areal velocity vector varied with submovement direction. Specifically,

$$\boldsymbol{v}_i = s_i .\boldsymbol{b}(\theta_i), \tag{4}$$

with the direction-dependent component composed of a Fourier series,

$$\boldsymbol{b}(\theta_i) = \boldsymbol{b}_0 + \boldsymbol{b}_1 \cos(\theta_i) + \boldsymbol{b}_2 \sin(\theta_i) + \boldsymbol{b}_3 \cos(2\theta_i) + \boldsymbol{b}_4 \sin(2\theta_i). \tag{5}$$

We included only terms up to $2\theta_i$ to prevent overfitting. The 15 free parameters were obtained by linear regression over the entire data set, excluding one submovement that was used for cross-validation.

(2) We assessed how well the model predicted the speed (or direction) of the excluded submovement from the magnitude (or orientation) of the areal velocity vector associated with that submovement. The decoded submovement speed was proportional to the magnitude of the areal velocity vector,

$$Decoded\ speed(\hat{s}_j) = \frac{|\boldsymbol{v}_j|}{|\boldsymbol{b}_0|}, \tag{6}$$

whereas the decoded submovement direction was that which minimized the angular deviation between actual and predicted areal velocity vectors, calculated by the vector dot product,

$$Decoded\ direction\left(\hat{\theta}_j\right) = \arg\max_\theta \left(\frac{\boldsymbol{v}_j .\boldsymbol{b}(\theta)}{|\boldsymbol{b}(\theta)|}\right). \tag{7}$$

Steps 1 and 2 were repeated with a different submovement excluded until the speed and direction of all N submovements had been estimated.

(3) We quantified decoding performance using coefficients of determination,

$$CoD(speed) = 1 - \frac{\sum_j (s_j - \hat{s}_j)^2}{\sum_j s_j^2} \tag{8}$$

determined from the isometric task recordings. Nevertheless, a consistent rotational structure is observed across all behavioral states. See also Movie S5.

(B) Summary of the preferred phase of neural firing within the M1 LFP cycle, relative to the LFP phase at peak movement speed. Data are for 98/125 (isometric task), 71/83 (free reaching), 61/78 (sleep), and 89/122 (sedation) neurons from M1/PMv, respectively. p values indicate the significance of the Rayleigh test of circular nonuniformity. Monkey R, open circles; monkey D, filled circles; monkey S, crosses. See also Figure S5.

$$CoD(direction) = \frac{1}{N}\sum_{j}\cos\left(\theta_j - \widehat{\theta}_j\right). \quad (9)$$

(4) Finally, we determined significance thresholds ($p < 0.05$) for CoD values by repeating the entire procedure for 1,000 surrogate data sets in which either the speed or direction was shuffled across submovements.

Phase of LFP Relative to Submovements and K Complexes

The phase of each LFP relative to submovements and K complexes was determined at the time of the event from a Hilbert transform of the event-triggered average. The correlation between LFP phase relative to each event was tested using the circular-circular correlation coefficient available in the CircStat toolbox (Berens, 2009).

2D LFP-PC Trajectory Model

The simplest linear system with oscillatory dynamics is a 2D state vector $\boldsymbol{x}(t)$ that evolves according to Equation 1. Under the conditions trace ($\boldsymbol{A}$) = 0 and det ($\boldsymbol{A}$) > 0, this system exhibits stable periodic solutions. We regressed the time derivative, $\dot{\boldsymbol{x}}$, against 2D LFP-PCs, $\boldsymbol{x}$, to find the three free parameters of $\boldsymbol{A}$ (the fourth is fixed by the trace constraint). Because the LFP-PCs during the isometric task are orthogonal, we expect the elements of matrix $\boldsymbol{A}$ to be zero on the diagonal. However, we did not impose this constraint during model fitting, because it will not necessarily hold when data from other conditions are projected onto the same PC axes. We measured the quality of fit to the LFP-PC derivative $\widehat{\dot{\boldsymbol{x}}(\boldsymbol{t})}$ in each case with a vector coefficient of determination defined by

$$CoD = 1 - \frac{\int \left|\widehat{\dot{\boldsymbol{x}}(\boldsymbol{t})} - \dot{\boldsymbol{x}}(\boldsymbol{t})\right|^2 dt}{\int |\dot{\boldsymbol{x}}(\boldsymbol{t})|^2 dt}, \quad (10)$$

where integration was performed over the entire recording. The same analysis was applied separately to LFP data that had been filtered into delta- (0–5 Hz) and beta- (10–30 Hz) frequency bands using four-pole, zero-phase digital Butterworth filters before mean referencing and PCA.

Spike-Triggered Average LFP-PC Trajectories

M1 and PMv LFPs were averaged from 200 ms before to 200 ms after each spike. The LFP averages were then projected onto the PC axes determined from the isometric task data and normalized by the standard deviation of the firing rate. To allow comparison across different data sets, the M1 LFP-PC plane was rotated such that the average submovement-triggered trajectory had zero phase at the moment of peak cursor speed, and the phase of the spike firing was measured relative to this. Significant phase locking across the population of neurons was assessed using the Rayleigh test for circular uniformity in the CircStat toolbox.

SUPPLEMENTAL INFORMATION

Supplemental Information includes five figures, five tables, and five movies and can be found with this article online at http://dx.doi.org/10.1016/j.neuron.2014.07.022.

ACKNOWLEDGMENTS

We thank J. Tulip and K. Nazarpour for assistance and S.N. Baker for discussion. This work was supported by the Wellcome Trust (086561), Medical Research Council (MR/G0802195/1, MR/K501396/1), and Engineering and Physical Sciences Research Council (EP/H051570/1).

Accepted: July 14, 2014
Published: August 14, 2014

REFERENCES

Amzica, F., and Steriade, M. (1997). The K-complex: its slow (<1-Hz) rhythmicity and relation to delta waves. Neurology *49*, 952–959.

Amzica, F., and Steriade, M. (1998). Electrophysiological correlates of sleep delta waves. Electroencephalogr. Clin. Neurophysiol. *107*, 69–83.

Amzica, F., Nuñez, A., and Steriade, M. (1992). Delta frequency (1–4 Hz) oscillations of perigeniculate thalamic neurons and their modulation by light. Neuroscience *51*, 285–294.

Antelis, J.M., Montesano, L., Ramos-Murguialday, A., Birbaumer, N., and Minguez, J. (2013). On the usage of linear regression models to reconstruct limb kinematics from low frequency EEG signals. PLoS ONE *8*, e61976.

Bansal, A.K., Vargas-Irwin, C.E., Truccolo, W., and Donoghue, J.P. (2011). Relationships among low-frequency local field potentials, spiking activity, and three-dimensional reach and grasp kinematics in primary motor and ventral premotor cortices. J. Neurophysiol. *105*, 1603–1619.

Berens, P. (2009). CircStat: a MATLAB toolbox for circular statistics. J. Stat. Softw. *31*, 1–20.

Bradberry, T.J., Gentili, R.J., and Contreras-Vidal, J.L. (2010). Reconstructing three-dimensional hand movements from noninvasive electroencephalographic signals. J. Neurosci. *30*, 3432–3437.

Buzsáki, G., Anastassiou, C.A., and Koch, C. (2012). The origin of extracellular fields and currents—EEG, ECoG, LFP and spikes. Nat. Rev. Neurosci. *13*, 407–420.

Carracedo, L.M., Kjeldsen, H., Cunnington, L., Jenkins, A., Schofield, I., Cunningham, M.O., Davies, C.H., Traub, R.D., and Whittington, M.A. (2013). A neocortical delta rhythm facilitates reciprocal interlaminar interactions via nested theta rhythms. J. Neurosci. *33*, 10750–10761.

Cash, S.S., Halgren, E., Dehghani, N., Rossetti, A.O., Thesen, T., Wang, C., Devinsky, O., Kuzniecky, R., Doyle, W., Madsen, J.R., et al. (2009). The human K-complex represents an isolated cortical down-state. Science *324*, 1084–1087.

Churchland, M.M., Cunningham, J.P., Kaufman, M.T., Foster, J.D., Nuyujukian, P., Ryu, S.I., and Shenoy, K.V. (2012). Neural population dynamics during reaching. Nature *487*, 51–56.

Colrain, I.M. (2005). The K-complex: a 7-decade history. Sleep *28*, 255–273.

Craik, K.J. (1947). Theory of the human operator in control systems; the operator as an engineering system. Br. J. Psychol. Gen. Sect. *38*, 56–61.

Destexhe, A., and Sejnowski, T.J. (2003). Interactions between membrane conductances underlying thalamocortical slow-wave oscillations. Physiol. Rev. *83*, 1401–1453.

Dine, J., Kühne, C., Deussing, J.M., and Eder, M. (2014). Optogenetic evocation of field inhibitory postsynaptic potentials in hippocampal slices: a simple and reliable approach for studying pharmacological effects on $GABA_A$ and $GABA_B$ receptor-mediated neurotransmission. Front. Cell. Neurosci. *8*, 2.

Dipietro, L., Poizner, H., and Krebs, H.I. (2011). EEG correlates of submovements. Conf. Proc. IEEE Eng. Med. Biol. Soc. *2011*, 7429–7432.

Doeringer, J.A., and Hogan, N. (1998). Intermittency in preplanned elbow movements persists in the absence of visual feedback. J. Neurophysiol. *80*, 1787–1799.

Grillner, S. (2006). Biological pattern generation: the cellular and computational logic of networks in motion. Neuron *52*, 751–766.

Guido, W., and Weyand, T. (1995). Burst responses in thalamic relay cells of the awake behaving cat. J. Neurophysiol. *74*, 1782–1786.

Hoshi, E., and Tanji, J. (2006). Differential involvement of neurons in the dorsal and ventral premotor cortex during processing of visual signals for action planning. J. Neurophysiol. *95*, 3596–3616.

Hwang, E.J., and Andersen, R.A. (2013). The utility of multichannel local field potentials for brain-machine interfaces. J. Neural Eng. *10*, 046005.

Jackson, A., and Fetz, E.E. (2007). Compact movable microwire array for long-term chronic unit recording in cerebral cortex of primates. J. Neurophysiol. *98*, 3109–3118.

Jerbi, K., Lachaux, J.P., N'Diaye, K., Pantazis, D., Leahy, R.M., Garnero, L., and Baillet, S. (2007). Coherent neural representation of hand speed in humans revealed by MEG imaging. Proc. Natl. Acad. Sci. USA *104*, 7676–7681.

Kiehn, O. (2006). Locomotor circuits in the mammalian spinal cord. Annu. Rev. Neurosci. *29*, 279–306.

Kunesch, E., Binkofski, F., and Freund, H.J. (1989). Invariant temporal characteristics of manipulative hand movements. Exp. Brain Res. *78*, 539–546.

Massey, J.T., Lurito, J.T., Pellizzer, G., and Georgopoulos, A.P. (1992). Three-dimensional drawings in isometric conditions: relation between geometry and kinematics. Exp. Brain Res. *88*, 685–690.

McAuley, J.H., Farmer, S.F., Rothwell, J.C., and Marsden, C.D. (1999). Common 3 and 10 Hz oscillations modulate human eye and finger movements while they simultaneously track a visual target. J. Physiol. *515*, 905–917.

Miall, R.C., and Jackson, J.K. (2006). Adaptation to visual feedback delays in manual tracking: evidence against the Smith Predictor model of human visually guided action. Exp. Brain Res. *172*, 77–84.

Miall, R.C., Weir, D.J., and Stein, J.F. (1986). Manual tracking of visual targets by trained monkeys. Behav. Brain Res. *20*, 185–201.

Miall, R.C., Weir, D.J., and Stein, J.F. (1993). Intermittency in human manual tracking tasks. J. Mot. Behav. *25*, 53–63.

Milner, T.E., and Ijaz, M.M. (1990). The effect of accuracy constraints on three-dimensional movement kinematics. Neuroscience *35*, 365–374.

Murphy, M., Riedner, B.A., Huber, R., Massimini, M., Ferrarelli, F., and Tononi, G. (2009). Source modeling sleep slow waves. Proc. Natl. Acad. Sci. USA *106*, 1608–1613.

Nauhaus, I., Busse, L., Carandini, M., and Ringach, D.L. (2009). Stimulus contrast modulates functional connectivity in visual cortex. Nat. Neurosci. *12*, 70–76.

Pasalar, S., Roitman, A.V., and Ebner, T.J. (2005). Effects of speeds and force fields on submovements during circular manual tracking in humans. Exp. Brain Res. *163*, 214–225.

Ray, S., and Maunsell, J.H. (2011). Network rhythms influence the relationship between spike-triggered local field potential and functional connectivity. J. Neurosci. *31*, 12674–12682.

Rickert, J., Oliveira, S.C., Vaadia, E., Aertsen, A., Rotter, S., and Mehring, C. (2005). Encoding of movement direction in different frequency ranges of motor cortical local field potentials. J. Neurosci. *25*, 8815–8824.

Riehle, A., Wirtssohn, S., Grün, S., and Brochier, T. (2013). Mapping the spatiotemporal structure of motor cortical LFP and spiking activities during reach-to-grasp movements. Front. Neural Circuits *7*, 48.

Roitman, A.V., Massaquoi, S.G., Takahashi, K., and Ebner, T.J. (2004). Kinematic analysis of manual tracking in monkeys: characterization of movement intermittencies during a circular tracking task. J. Neurophysiol. *91*, 901–911.

Rubino, D., Robbins, K.A., and Hatsopoulos, N.G. (2006). Propagating waves mediate information transfer in the motor cortex. Nat. Neurosci. *9*, 1549–1557.

Ruspantini, I., Saarinen, T., Belardinelli, P., Jalava, A., Parviainen, T., Kujala, J., and Salmelin, R. (2012). Corticomuscular coherence is tuned to the spontaneous rhythmicity of speech at 2–3 Hz. J. Neurosci. *32*, 3786–3790.

Sailer, U., Flanagan, J.R., and Johansson, R.S. (2005). Eye-hand coordination during learning of a novel visuomotor task. J. Neurosci. *25*, 8833–8842.

Schöner, G., and Kelso, J.A. (1988). Dynamic pattern generation in behavioral and neural systems. Science *239*, 1513–1520.

Selen, L.P., van Dieën, J.H., and Beek, P.J. (2006). Impedance modulation and feedback corrections in tracking targets of variable size and frequency. J. Neurophysiol. *96*, 2750–2759.

Shenoy, K.V., Sahani, M., and Churchland, M.M. (2013). Cortical control of arm movements: a dynamical systems perspective. Annu. Rev. Neurosci. *36*, 337–359.

Steriade, M., and Llinás, R.R. (1988). The functional states of the thalamus and the associated neuronal interplay. Physiol. Rev. *68*, 649–742.

Umilta, M.A., Brochier, T., Spinks, R.L., and Lemon, R.N. (2007). Simultaneous recording of macaque premotor and primary motor cortex neuronal populations reveals different functional contributions to visuomotor grasp. J. Neurophysiol. *98*, 488–501.

Waldert, S., Preissl, H., Demandt, E., Braun, C., Birbaumer, N., Aertsen, A., and Mehring, C. (2008). Hand movement direction decoded from MEG and EEG. J. Neurosci. *28*, 1000–1008.

Waldert, S., Pistohl, T., Braun, C., Ball, T., Aertsen, A., and Mehring, C. (2009). A review on directional information in neural signals for brain-machine interfaces. J. Physiol. Paris *103*, 244–254.

Neuron, Volume *83*

Supplemental Information

A Common Structure Underlies Low-Frequency Cortical Dynamics in Movement, Sleep, and Sedation

Thomas M. Hall, Felipe de Carvalho, and Andrew Jackson

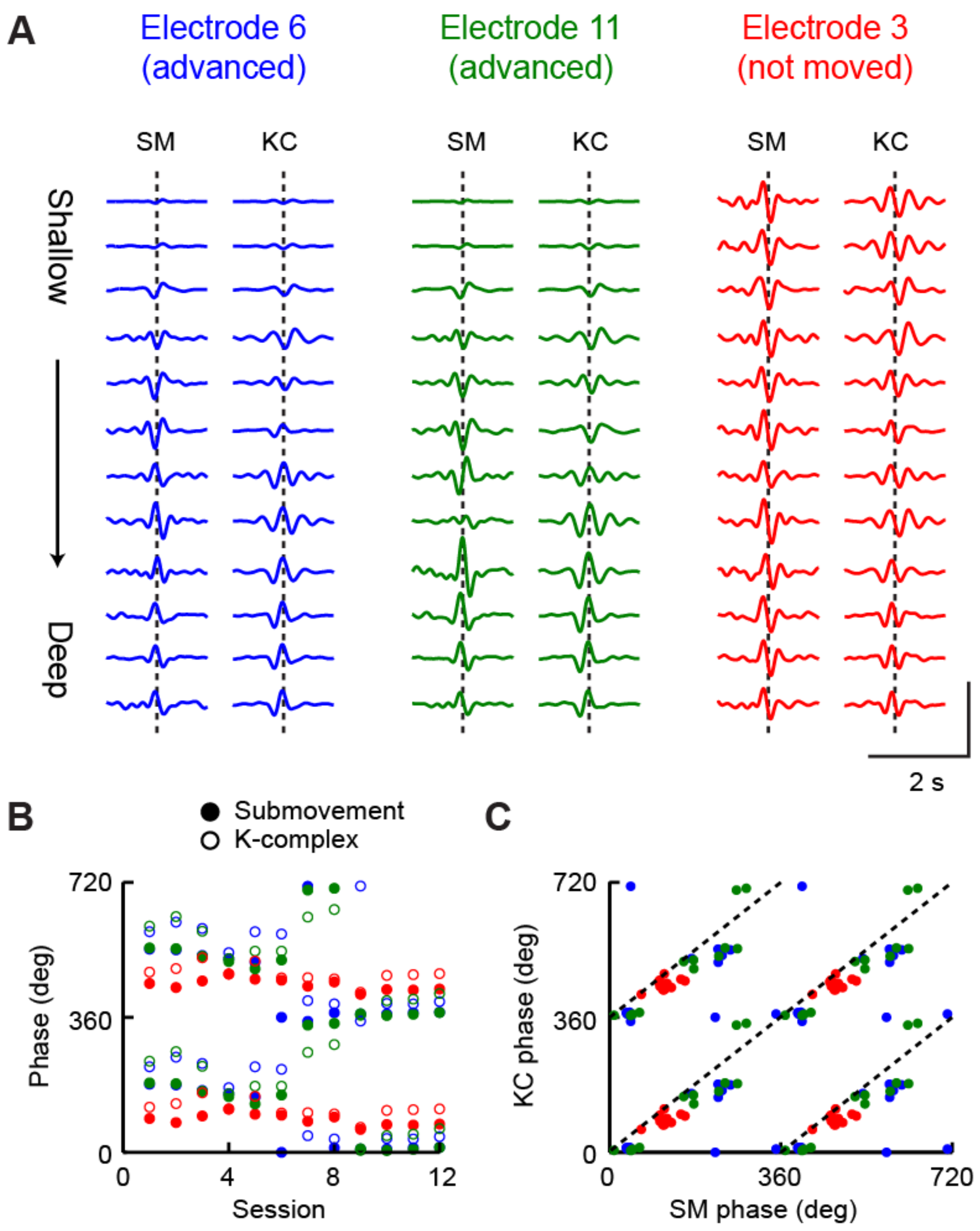

Figure S1 Related to Figure 4: Submovement- and K-complex-related LFPs share common cortical sources.

(A) Over progressive days (alternating between awake and sedated recordings), electrodes 6 and 11 were advanced from the cortical surface down through the gray matter in steps of approximately 0.5 mm. Electrode 3 was left at a fixed depth as a control. Plot shows average LFPs from these electrodes aligned to submovements (SM) and K-complexes (KC). Vertical scale: 40 μV (SM), 1000 μV (KC).

(B) Event-triggered LFP-phase for submovements (filled circles) and K-complexes (open circles) over successive sessions. Note the 180° phase shift (polarity reversal) that occurs in both submovement- and KC-triggered averages for electrode 6 (blue) and electrode 11 (green) between sessions 5-7, corresponding to a depth of approximately 3 mm. At this location we were able to record large single-units (not shown) on the same electrodes, indicating polarity reversal occurred within the grey matter. Electrode 3 (red) was not moved and no polarity inversion is seen.

(C) KC-phase plotted against SM-phase for each electrode over successive sessions. Points are color-coded according to electrode number. Across all depths the phase relative to submovements was strongly correlated with phase relative to K-complexes ($n=36$, $\rho_{CC}=0.75$, $P=8\times10^{-5}$).

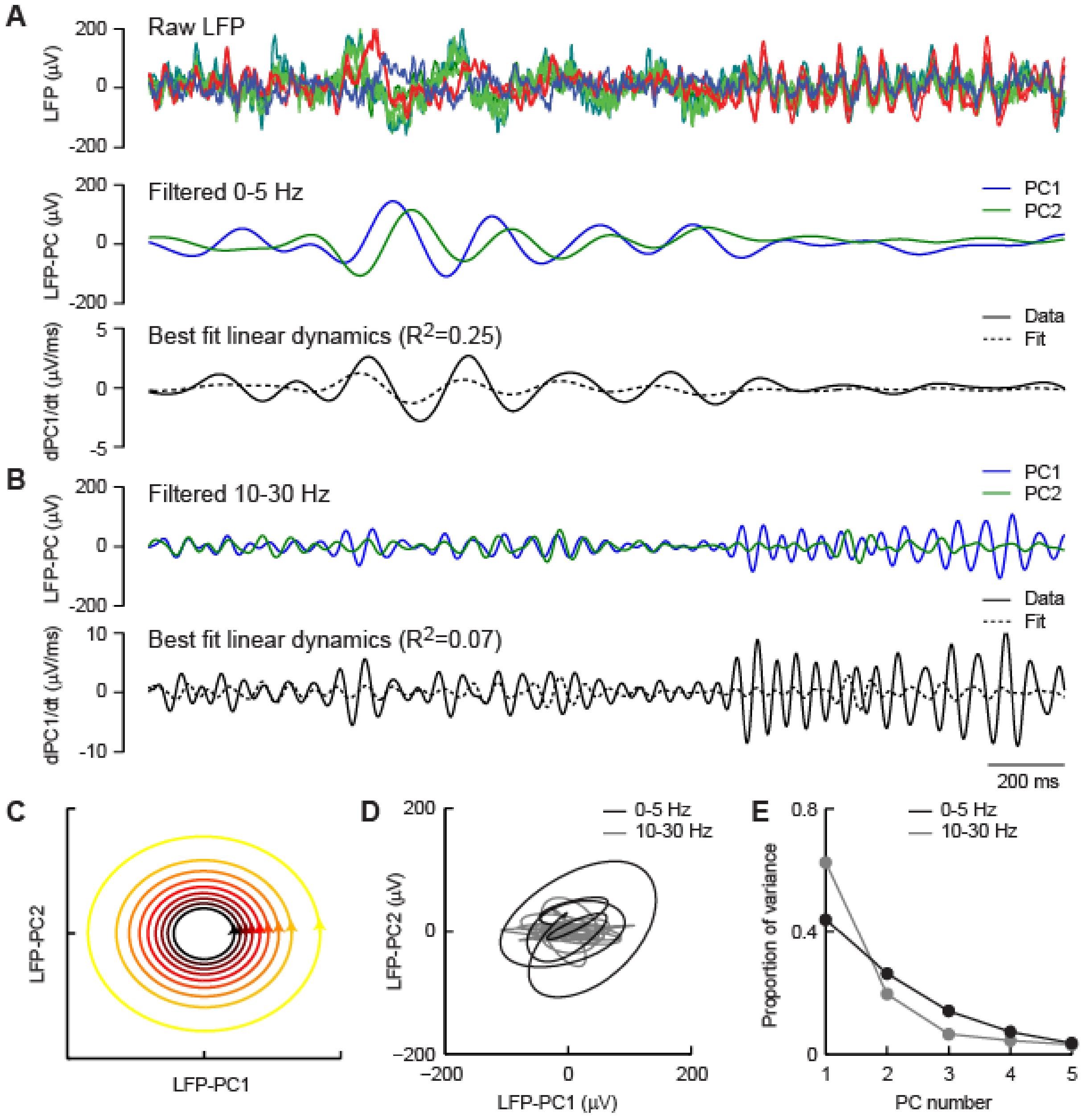

Figure S2 Related to Figure 5: A linear dynamical model fitted to LFP data in the delta and beta band.

(A) *Top:* Section of unfiltered, multichannel LFP from 9 M1 electrodes during task performance exhibiting delta and beta band oscillation. *Middle:* LFP-PCs calculated for LFP data filtered in the delta band (0-5 Hz). *Bottom:* Model fit of the time-derivative of LFP-PC1 from weighted sum of LFP-PC1 and LFP-PC2. Note that due to the consistent 90° phase-shift between PCs, the time-derivative of LFP-PC1 closely matches the negation of LFP-PC2.

(B) Equivalent analysis for same LFP data filtered in the beta band (10-30 Hz). Note that due to the lack of a consistent relationship between LFP-PCs, the time-derivative of LFP-PC1 cannot be well described by a weighted sum of LFP-PC1 and LFP-PC2.

(C) Sample closed trajectories of linear model fit to delta band LFP data.

(D) Example real LFP-PC trajectories for delta band (black) and beta band (grey).

(E) Proportion of variance captured by first five PCs in delta (black) and beta (grey) band. Note that the beta band predominantly comprises a single component (explaining ~60% of the variance), whereas the delta band signal is distributed across multiple components.

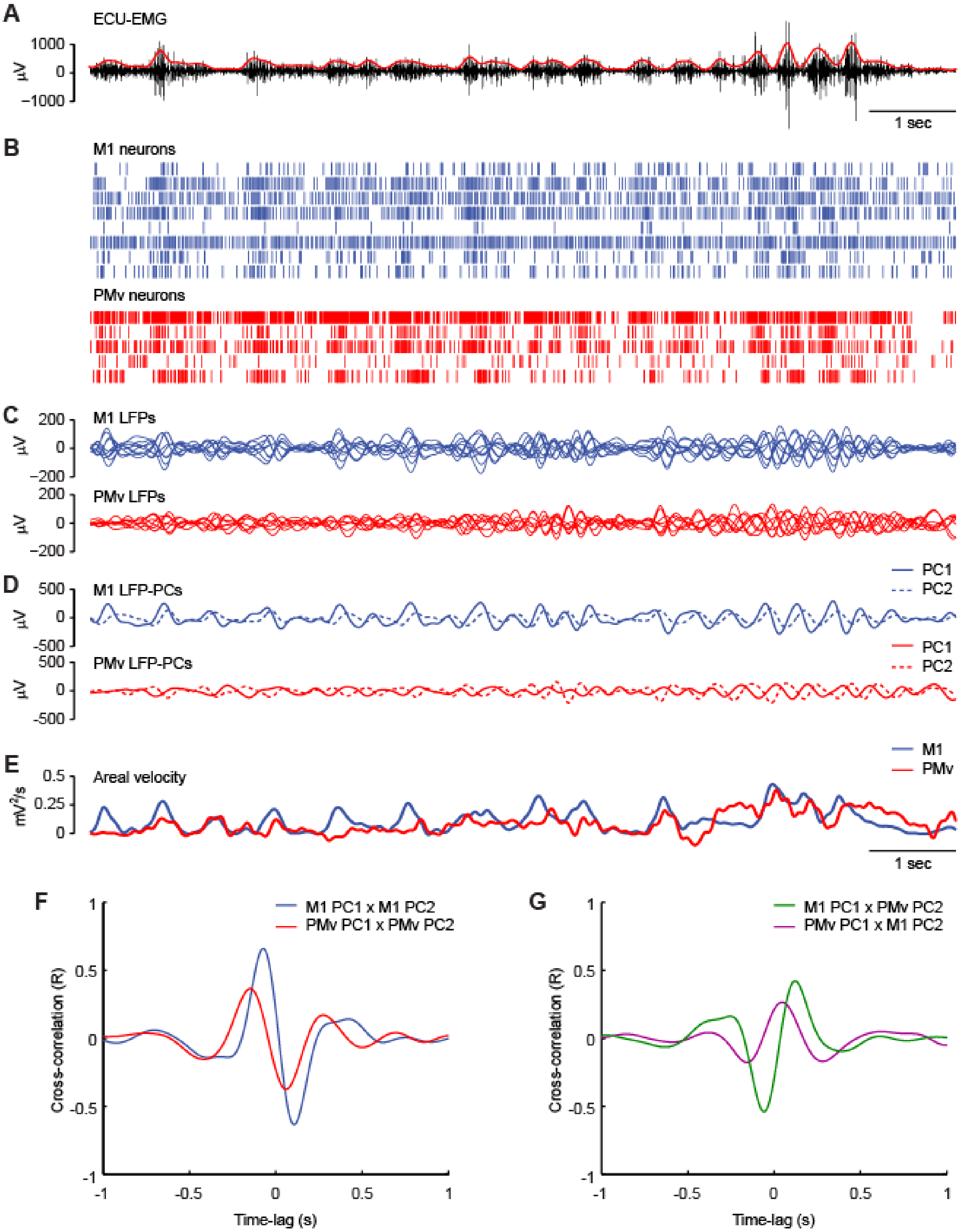

Figure S3 Related to Figure 6: M1 and PMv are co-active during free reach-to-grasp.

(A) EMG recording from *extensor carpi radialis* muscle as monkey D retrieved food rewards from small wells in a Klüver board with the arm contralateral to recording sites.

(B) Spike rasters for 8 neurons in M1 (blue) and 6 neurons in PMv (red). Note that neurons in both areas are co-active with EMG in the contralateral limb.

(C) Low-pass filtered, mean-subtracted LFPs recorded from 10 electrodes in M1 and 8 electrodes in PMv show bursts of low-frequency oscillation.

(D) LFP-PCs calculated from M1 and PMv recordings.

(E) Areal velocity in the PC plane for M1 and PMv LFPs.

(F) Cross-correlation between LFP-PCs within the same cortical area.

(G) Cross-correlation between LFP-PCs across cortical areas.

Data from monkey D, recorded at the end of the task session shown in Figure 6.

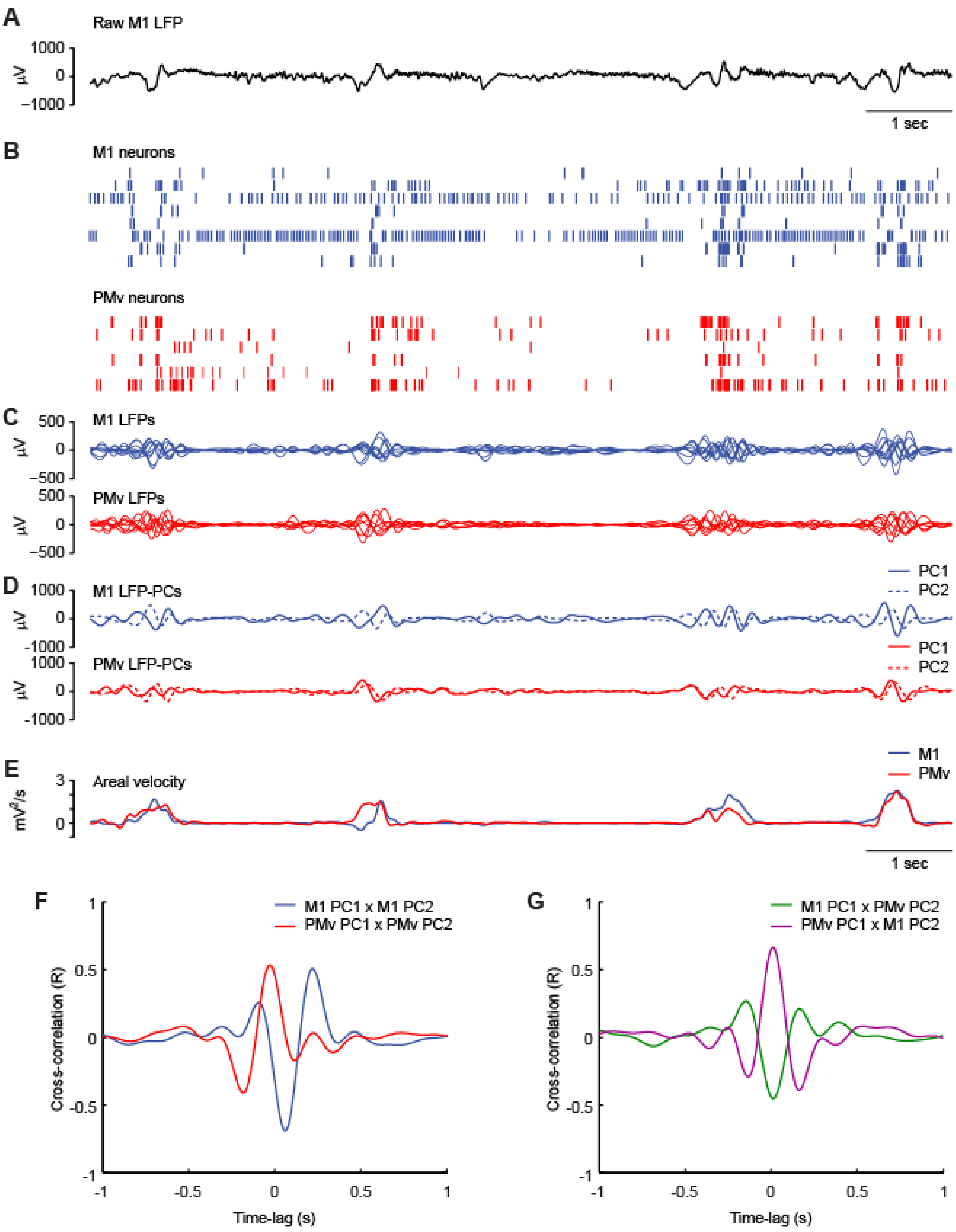

Figure S4 Related to Figure 6: M1 and PMv are co-active during K-complexes under ketamine sedation.

(A) Unfiltered LFP recording from M1 during ketamine sedation showing intermittent K-complexes.

(B) Spike rasters for 8 neurons in M1 (blue) and 6 neurons in PMv (red). Note that neurons in both areas are quiet immediately preceding the K-complex (down state) and fire maximally during its rising phase and peak (up state).

(C) Low-pass filtered, mean-subtracted LFPs recorded from 10 electrodes in M1 and 8 electrodes in PMv show bursts of low-frequency oscillation around K-complexes.

(D) LFP-PCs calculated from M1 and PMv recordings.

(E) Areal velocity in the PC plane for M1 and PMv LFPs.

(F) Cross-correlation between LFP-PCs within the same cortical area.

(G) Cross-correlation between LFP-PCs across cortical areas.

Data from monkey D, recorded following the session shown in Figure 6.

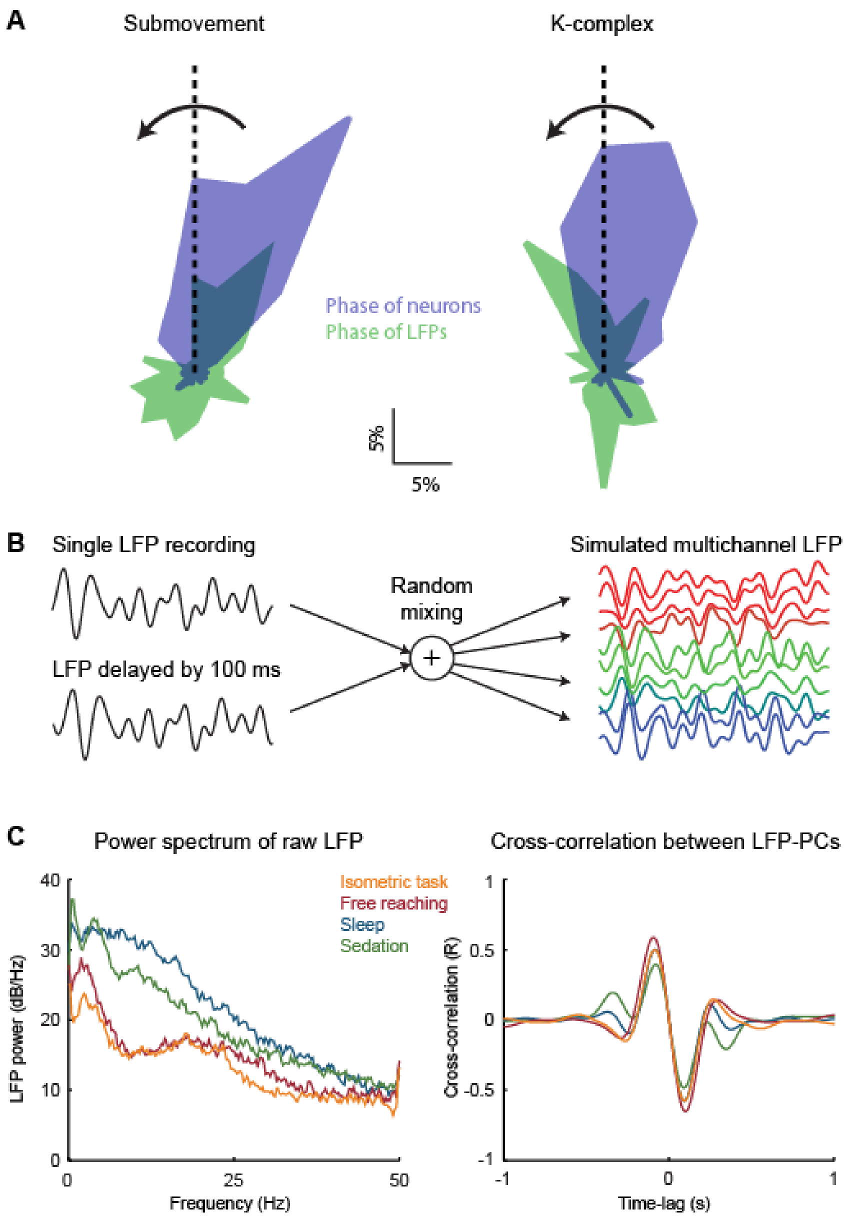

Figure S5 Related to Figure 8: Multiple phases of low-frequency oscillation are present in LFPs, while neurons fire predominantly at a single phase.

(A) Phase histogram for M1 neuronal firing (blue) and LFP (green) relative to submovements (*left*) and K-complexes (*right*). LFP data is taken from Figure 4F. Neuronal data is adapted from Figure 8. Note that the majority of M1 cells are active at a phase that slightly precedes the peak submovement speed or K-complex. By contrast, the phase of the LFP exhibits a more uniform distribution relative to each event.

(B) A simple model to explain how multiple LFP phases can arise from a mixture of underlying sources with fixed temporal delay. In this case, two LFP sources have a relative time-lag of 100 ms. Multiple LFPs are simulated by different linear mixtures of the two underlying sources.

(C) The model applied to real data. The power spectrum (*left*) is for a single channel of LFP (as in Fig. 5A). This channel was used to generate two sources, from which 10 random mixtures were drawn to simulate multichannel LFP recordings. PCA was applied to the simulated data, and the first two LFP-PCs exhibited a consistent correlation structure (*right*) that was qualitatively similar to the real data (Fig. 5B). Note that this result is relatively insensitive to the precise time delay (in this case 100 ms), since PCA finds orthogonal projections of the underlying oscillation. We speculate that the LFP captures a component of population activity that has a consistent phase-lag relative to neuronal firing. Such a component could reflect inhibitory neurons that are under-sampled in our recordings, due to their size and/or location. Alternatively, the delayed LFP component could reflect slow $GABA_B$-mediated recurrent inhibition, or synaptic currents arising from thalamo-cortical loops. Further research will be required to distinguish these possibilities.

**Monkey**	**Number of electrodes**	**Number of submovements**	**CoD** (speed)	**CoD** (direction)
D	10	1715	**0.46**	**0.07**
D	10	2043	**0.24**	0.04
D	10	1724	**0.49**	**0.17**
D	10	2049	**0.48**	**0.24**
D	10	1515	**0.45**	**0.12**
D	10	2272	**0.34**	**0.13**
R	10	914	**0.31**	**0.32**
R	10	1259	**0.04**	**0.16**
R	10	501	**0.27**	**0.23**
R	9	808	**0.22**	**0.15**
S	7	627	**0.21**	**0.12**
S	7	933	**0.17**	**0.08**
S	7	396	**0.25**	**0.12**
Average ± SD			*0.30 ± 0.13*	*0.15 ± 0.07*

Table S1 Related to Figure 2: Decoding speed and direction of individual submovements from the magnitude and direction of areal velocity vector in the 3D LFP-PC space. The average areal velocity vectors associated with all but one submovement were fitted with a parametric function of submovement speed and direction (Equ. 4,5; Experimental procedures). The speed (or direction) of the excluded submovement was then decoded from the magnitude (or orientation) of its associated areal velocity vector (Equ. 6,7). This was procedure was repeated for all submovements in the session, and the quality of the decoding was assessed using scalar (or angular) CoD (Equ. 8,9) which ranges from 0 (chance performance) to 1 (perfect decoding). The significance of individual CoD values was assessed using surrogate datasets in which the speed (or direction) was shuffled across submovements. Bold indicates P<0.05.

**Monkey**	**Number of electrodes**	**ρ_{CC}**
D	11	**0.63**
D	10	0.06
D	11	**0.84**
D	10	**0.85**
D	10	**0.81**
R	9	**0.90**
R	9	**0.95**
R	9	**0.86**
R	9	0.46
S	11	**0.95**
S	11	**0.93**
S	11	**0.93**
S	12	**0.56**
Average ± SD		*0.75 ± 0.25*

Table S2 Related to Figure 4. Relationship between M1 LFP phase relative to submovements (during task performance) versus phase relative to K-complexes (under sedation). Event-triggered average LFP traces were compiled for each LFP electrode separately. The LFP phase relative to each event (submovement or K-complex) was determined using a Hilbert transform. The relationship between these phases across all electrodes was quantified using the circular-circular correlation coefficient (ρ_{CC}). Bold indicates $P<0.05$.

Monkey	Delta band (0-5 Hz)			Beta band (10-30 Hz)		
	Task fit	Task-sedation generalization	Sedation fit	Task fit	Task-sedation generalization	Sedation fit
D	0.31	0.41	0.50	0.14	-0.08	0.02
D	0.33	0.28	0.44	0.02	0.04	0.07
D	0.22	0.26	0.43	0.03	-0.05	0.002
D	0.31	0.14	0.32	0.13	-0.08	0.08
D	0.22	0.07	0.15	0.13	-0.09	0.10
R	0.14	0.03	0.04	0.01	0.01	0.007
R	0.27	0.27	0.27	0.01	0.01	0.008
R	0.09	0.12	0.16	0.06	-0.11	0.0009
R	0.15	0.12	0.14	0.08	-0.11	0.003
S	0.13	0.16	0.18	0.01	-0.02	0.001
S	0.17	0.24	0.25	0.06	-0.11	0.001
S	0.16	0.23	0.26	0.04	-0.05	0.002
Average (SD)	*0.20 (0.08)*	*0.20 (0.10)*	*0.26 (0.13)*	*0.06 (0.05)*	*-0.05 (0.05)*	*0.02 (0.03)*

Table S3 Related to Figure 5: Linear dynamical model fit to 2D LFP-PC data in delta and beta bands for task performance and ketamine sedation. For each frequency band, the first column shows quality of fit (measured by CoD; Equ. 10) when model parameters were fitted to and tested on the same data, recorded during performance of the isometric task (Task fit). The second column shows how well the same model parameters that best-fit the task data were able to fit the data recorded on a different day during ketamine sedation (Task-sedation generalization). The third column shows the performance of the model using parameters that best-fit the sedation data when tested on the same data (Sedation fit). The data are plotted in Figure 5D.

Monkey	Delta band (0-5 Hz)			Beta band (10-30 Hz)		
	Task fit	Task-sleep generalization	Sleep fit	Task fit	Task-sleep generalization	Sleep fit
D	0.26	0.11	0.14	0.14	-0.11	0.03
D	0.24	0.24	0.26	0.14	-0.12	0.04
D	0.28	0.15	0.21	0.13	-0.13	0.02
D	0.22	0.17	0.18	0.13	0.01	0.09
S	0.18	0.15	0.18	0.02	-0.01	0.006
S	0.13	0.20	0.22	0.01	0.05	0.09
S	0.17	0.12	0.14	0.06	0.11	0.13
S	0.16	0.13	0.14	0.04	0.09	0.11
S	0.15	0.11	0.12	0.03	0.07	0.09
Average (SD)	*0.20 (0.05)*	*0.15 (0.04)*	*0.18 (0.04)*	*0.08 (0.05)*	*-0.004 (0.09)*	*0.07 (0.04)*

Table S4 Related to Figure 5: Linear dynamical model fit to 2D LFP-PC data in delta and beta bands for task performance and natural sleep. For each frequency band, the first column shows quality of fit (measured by CoD; Equ. 10) when model parameters were fitted to and tested on the same data, recorded during performance of the isometric task (Task fit). The second column shows how well the same model parameters that best-fit the task data were able to fit the data recorded during natural sleep at the end of the session (Task-sleep generalization). The third column shows the performance of the model using parameters that best-fit the sleep data when tested on the same data (Sleep fit). The data are plotted in Figure 5E.

Monkey	Delta band (0-5 Hz)			Beta band (10-30 Hz)		
	Task fit	Task-free generalization	Free fit	Task fit	Task-free generalization	Free fit
D	0.33	0.17	0.23	0.11	0.09	0.10
D	0.26	0.25	0.30	0.16	0.14	0.14
D	0.31	0.16	0.25	0.15	0.11	0.11
D	0.28	0.17	0.26	0.13	0.12	0.12
D	0.22	0.20	0.25	0.13	0.11	0.12
D	0.23	0.30	0.39	0.14	0.12	0.13
S	0.13	0.14	0.14	0.03	0.01	0.01
S	0.12	0.13	0.14	0.04	0.01	0.02
S	0.11	0.15	0.16	0.02	0.02	0.02
S	0.11	0.15	0.16	0.02	0.01	0.01
Average (SD)	*0.21 (0.08)*	*0.18 (0.05)*	*0.23 (0.08)*	*0.09 (0.05)*	*0.05 (0.05)*	*0.08 (0.05)*

Table S5 Related to Figure 5: Linear dynamical model fit to 2D LFP-PC data in delta and beta bands for task performance and free reaching for food in a Klüver board. For each frequency band, the first column shows quality of fit (measured by CoD; Equ. 10) when model parameters were fitted to and tested on the same data, recorded during performance of the isometric task (Task fit). The second column shows how well the same model parameters that best-fit the task data were able to fit the data recorded during free reaching at the end of the session (Task-free generalization). The third column shows the performance of the model using parameters that best-fit the free reaching data when tested on the same data (Free fit). The data are plotted in Figure 5F.

Supplementary Movies

Movie S1 Related to Figure 1: Sample M1 LFP recordings and M1 LFP-PC trajectories during torque tracking in real-time. *Top left*: task display with cursor (yellow) and target (red). *Bottom*: low-pass filtered LFP data. *Top right*: Instantaneous LFP-PCs projected onto the 2D plane.

Movie S2 Related to Figure 2: Animation of submovement-triggered average 2D LFP-PC trajectories divided according to peak cursor speed for monkey D and monkey R. Trajectories are shown from 200ms before to 200ms after the midpoint of each submovement (indicated by filled circles). Trials are sorted and color-coded according to cursor speed, as in Fig. 2F,I. Animation runs three times.

Movie S3 Related to Figure 2: Animation of submovement-triggered average 3D LFP-PC trajectories divided according to submovement direction. Trajectories are shown from 200ms before to 200ms after the midpoint of each submovement (indicated by filled circles). Trials are sorted and color-coded according to direction of movement, as in Fig. 2L,M. Thick lines indicate orientation of areal velocity vectors.

Movie S4 Related to Figure 5: Motor cortical LFP dynamics under different wake/sleep/sedation states in real-time. *Right*: Low-pass filtered LFP data in real-time during isometric movement, natural sleep and ketamine sedation. *Left*: Instantaneous projection onto PC plane (determined from data recorded during task performance). Note that the axes for movement data have been expanded x4 to compensate for the smaller amplitude of movement-related LFP relative to sleep and sedation.

Movie S5 Related to Figure 8: Animation of spike-triggered average of LFP-PC trajectories. Trajectories are shown from 200ms before to 200ms after the time of spikes from M1 (blue) and PMv (red), as in Fig. 8. Top row shows M1 LFPs and bottom row shows PMv LFPs. Different columns correspond to different behavioural states (isometric task, free reaching, natural sleep and ketamine sedation). In all cases the data are projected onto the PC plane determined from recordings during task performance. Animation runs three times.
